# Avian Influenza A Virus Infection among Workers at Live Poultry Markets, China, 2013–2016

**DOI:** 10.3201/eid2407.172059

**Published:** 2018-07

**Authors:** Mai-Juan Ma, Teng Zhao, Shan-Hui Chen, Xian Xia, Xiao-Xian Yang, Guo-Lin Wang, Li-Qun Fang, Guan-Yuan Ma, Meng-Na Wu, Yan-Hua Qian, Natalie E. Dean, Yang Yang, Bing Lu, Wu-Chun Cao

**Affiliations:** Beijing Institute of Microbiology and Epidemiology, Beijing, China (M.-J. Ma, T. Zhao, X. Xia, X.-X. Yang, G.-L. Wang, L.-Q. Fang, M.-N. Wu, W.-C. Cao);; Wuxi Municipal Center for Disease Control and Prevention, Wuxi, China (S.-H. Chen, G.-Y. Ma, Y.-H. Qian, B. Lu);; The PLA Army General Hospital, Beijing (X. Xia);; Taihu University of Wuxi, Wuxi (X.-X. Yang);; University of Florida, Gainesville, Florida, USA (N.E. Dean, Y. Yang)

**Keywords:** Avian influenza virus, incidence, influenza A virus, live poultry markets, poultry workers, viruses, China, reassortment, risk factors, seroconversion, seroprevalence, surveillance, zoonoses, influenza

## Abstract

We conducted a 3-year longitudinal serologic survey on an open cohort of poultry workers, swine workers, and general population controls to assess avian influenza A virus (AIV) seroprevalence and seroincidence and virologic diversity at live poultry markets (LPMs) in Wuxi City, Jiangsu Province, China. Of 964 poultry workers, 9 (0.93%) were seropositive for subtype H7N9 virus, 18 (1.87%) for H9N2, and 18 (1.87%) for H5N1. Of 468 poultry workers followed longitudinally, 2 (0.43%), 13 (2.78%), and 7 (1.5%) seroconverted, respectively; incidence was 1.27, 8.28, and 4.46/1,000 person-years for H7N9, H9N2, and H5N1 viruses, respectively. Longitudinal surveillance of AIVs at 9 LPMs revealed high co-circulation of H9, H7, and H5 subtypes. We detected AIVs in 726 (23.3%) of 3,121 samples and identified a high diversity (10 subtypes) of new genetic constellations and reassortant viruses. These data suggest that stronger surveillance for AIVs within LPMs and high-risk populations is imperative.

Avian influenza A viruses (AIVs) remain an important threat to human health. With new strains widely circulating in China, an increasing number of human infections with AIVs have been reported since 2013, including subtypes H7N9, H5N6, and H10N8 (*1*–*3*). In addition, more human infections with H9N2 have been reported since 2014 (*4*). Although no sustained human-to-human transmission has been observed for these viral subtypes, serious concern exists that the virus could become more efficient in causing human epidemics (*5*).

Most human infections with AIVs (e.g., subtypes H7N9, H5N1, and H5N6) have been associated with exposure to poultry and resulted in severe illness (*6*). However, these severely ill patients could represent the tip of the iceberg because mild and asymptomatic infections with H7N9, H9N2, and H5N1 subtypes have been observed by surveillance (*7*–*11*) and serologic studies (*12*–*18*). Surveillance might miss persons with mild or asymptomatic infection who do not seek medical care. Cross-sectional serologic studies have limited value for measuring incidence rates of AIV infections, resulting in poor understanding of the prevalence of infection and the proportion of cases that are mild or subclinical in humans.

The southern provinces of China have a high density of poultry and humans and are considered likely hot spots for the emergence of new reassortant influenza viruses (*19*). China’s Jiangsu Province, one of the hot spots, has reported human infections with H7N9 and H5N1 subtypes. We conducted a 3-year longitudinal serologic study to estimate the seroprevalence and seroincidence of H7N9, H9N2, H5N1, and H5N6 subtypes among animal (poultry and swine) workers and general population controls and to identify the risk factors for seropositivity or seroconversion. We also conducted longitudinal surveillance to measure the diversity and genetic variation of AIVs at live poultry markets (LPMs) in the city of Wuxi, Jiangsu Province, China.

## Materials and Methods

### Study Population, Sampling, and Data Collection

During July 2013–September 2016, we conducted a longitudinal serologic survey among an open cohort of poultry and swine workers and general population controls in Wuxi. We recruited workers who were >18 years of age and were exposed to poultry and pigs or to poultry and pig manure as part of their daily activities (e.g., husbandry, slaughtering, sales). In addition, we recruited control participants from residents at community service centers who reported having no exposure to poultry or pigs or to animal manure as part of their daily activities. After enrolling participants in July 2013, we conducted follow-up visits at 1, 2, and 3 years. Because poultry and swine workers in China are often temporarily employed and different workers might be present each year, prospective follow-up of the same persons over the study period was not always feasible. Therefore, we enrolled new participants at each follow-up visit to maintain the number of active cohort participants at ≈2,000.

At participant enrollment, we used a comprehensive questionnaire to collect demographic data, exposure variables, information about any history of chronic medical conditions, influenza vaccination history, self-reported influenza-like illness during the past 12 months, and the extent and nature of exposure to animals or animal manure. At each follow-up visit, we used a shorter questionnaire to collect additional demographic data, recent history of exposure to poultry or pigs, and self-reported recent influenza-like illness. At enrollment and follow-up visits, we asked each participant to provide a 5-mL blood sample.

We obtained written informed consent from all participants before conducting interviews and collecting samples. The institutional review boards of the Beijing Institute of Microbiology and Epidemiology (no number given) approved the study protocol.

### Poultry and Environmental Surveillance of AIVs

**Figure 1:**
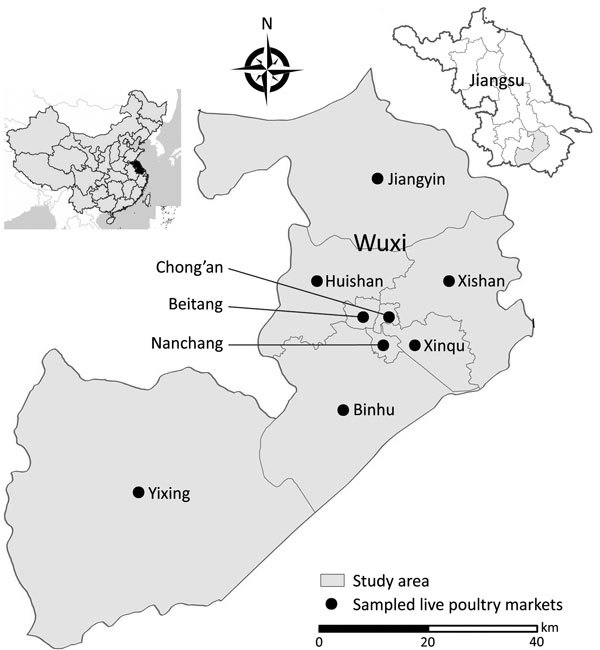
Location of study area where participants were enrolled and of live poultry markets where environmental and cloacal swab sampling was conducted in study of influenza A infection among workers at live poultry markets in 9 districts of Wuxi, Jiangsu Province, China, 2013–2016. Insets show location of Wuxi in Jiangsu Province and location of the province in China.

During the serologic study period, we also conducted prospective surveillance of AIVs at 9 LPMs in 9 districts of Wuxi (Figure 1). Once each month, we collected ≈54 cloacal swab samples (6 samples from each LPM) from chickens, ducks, or geese and preserved each sample in a tube containing 3 mL of viral transport medium (MT0301; Yocon, Beijing, China). In addition, 18 of each type of environmental swab and fecal/slurry samples were collected (2 samples of each type from each LPM). We collected environmental samples by swabbing surfaces of chicken epilators, chopping boards, cages, and sewage 4–8 times with separate cotton-tipped swabs. We then inserted the swabs into a tube containing 3 mL of viral transport medium (Yocon). Fecal (1 g) or slurry (1 mL) samples were collected at available sites and were diluted in viral transport medium (Yocon).

### Sample Processing and Laboratory Analysis

All blood, cloacal, and environmental specimens were kept on frozen cold packs at 2°C–8°C after collection and transported to the local Center for Disease Control and Prevention laboratory. Serum was separated by centrifugation for 5 min at 2,000 rpm. Cloacal and environmental specimens were vortexed, and swabs were discarded. For the fecal/slurry specimens, we conducted an extra centrifugation for 5 min at 2,000 rpm to separate the mixture of virus and viral medium. Each type of specimen was split into 3 aliquots and frozen at −80°C until use.

We first screened all serum samples by hemagglutination inhibition (HI) assay (*20*), and samples with an HI titer >10 were tested by a microneutralization (MN) assay (*21*). Considering the prevalence of avian-lineage viruses in China and their availability, we used a human H7N9 isolate (A/Jiangsu/Wuxi05/2013), clade 2.3.4.4 H5N6 virus (A/chicken/Jiangsu/WXBING2/2014), clade 2.3.2.1c H5N1 virus (A/chicken/Jiangsu/WX927/2013), and Y280-like H9N2 virus (A/chicken/Jiangsu/WXWA021/2013) for HI and MN assays. We defined a seropositive result as an MN titer >80 for all tested viruses. Seroconversion was defined as detection of a >4-fold rise in MN antibody titer between initial serum sample and a paired second serum sample, with the second sample achieving a titer >80. Additional details for the HI and MN assays, PCR detection, viral isolation, sequencing of the cloacal and environmental samples, and the phylogenetic analysis of the AIVs we identified are available in the Technical Appendix. We deposited sequence data in the GISAID database (http://platform.gisaid.org; accession nos. EPI_ISL_277027–277050, 277052–277064, and 277093–277127).

### Statistical Analysis

We calculated the proportion (and associated 95% CIs) of poultry workers, swine workers, and control participants who were seropositive or seroconverted. We estimated the incidence of seroconversion per 1,000 person-years (and associated 95% CIs) for participants with multiple longitudinal serum samples using the time between baseline and follow-up as their person-time contribution. We excluded participants who were seropositive at baseline. We analyzed categorical and continuous variables using the χ^2^ or Fisher exact test and the Student *t*-test where necessary. Risk factors for virus infection (any seropositivity or seroconversion for each individual) were assessed only among participants with paired serum samples using logistic regression models after adjustment for sex and age group or variables with p values <0.05, summarized by odds ratios (ORs) with 95% CIs. Exact Poisson regression model was used to explore the effect of exposure on 1,000 person-year incidence in the cohorts, assessed by incidence rate ratios with 95% CIs. All tests were 2-sided with a 0.05 level of significance. Analyses were performed using SPSS software version 16.0 (SPSS, Chicago, IL, USA).

## Results

### Demographic Characteristics of Participants

**Figure 2:**
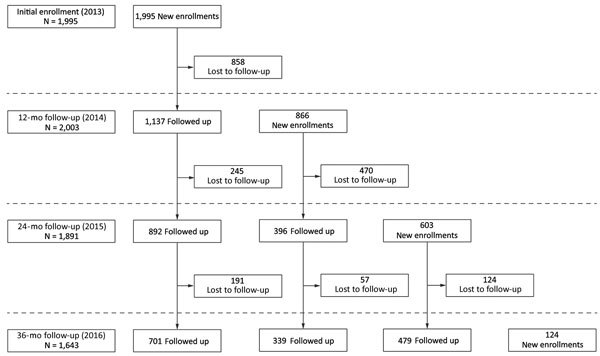
Flowchart for participant enrollment and follow-up in study of avian influenza A virus infection among workers at live poultry markets, Wuxi, Jiangsu Province, China, 2013–2016.

In July 2013, we enrolled 1,995 participants: 511 poultry workers, 569 swine workers, and 915 general population controls. Of these original 1,995 participants, 1,137 were followed up at year 1 (July 2014), 892 at year 2 (July 2015), and 701 at year 3 (July 2016) (Figure 2). To compensate for the number of participants lost to follow-up, we enrolled an additional 866 participants in July 2014, 603 in July 2015, and 124 in July 2016 (Figure 2). New participants enrolled in 2014 were also followed in 2015 (396) and 2016 (339) and new participants enrolled in 2015 were followed in 2016 (479) (Figure 2). Poultry and swine workers tended to be older and less educated than controls (p<0.05), and swine workers comprised a significantly higher proportion of men among the 3 groups (online Technical Appendix Table 1).

**Table 1:** Seroprevalence of microneutralization titers against influenza A(H7N9), A(H9N2), and A(H5N1) viruses in poultry workers, swine workers, and controls, eastern China, 2013–2016*

Antigen/year	No. seropositive/no. total (% [95% CI])			
Poultry workers	Swine workers	Controls	p value
H7N9				
2013	3/511 (0.58 [0.12–1.71])	0/569 (0 [0–0.65])	2/915 (0.22 [0.03–0.79])	0.13
2014	3/533 (0.56 [0.12–1.64])	1/589 (0.17 [0–0.94])	1/881 (0.11 [0–0.63])	0.19
2015	3/535 (0.56 [0.12–1.63])	0/501 (0 [0–0.73])	0/855 (0 [0–0.43])	0.04
2016	0/491 (0 [0–0.75])	1/367 (0.27 [0.01–1.51])	1/785 (0.13 [0–0.71])	0.48
Overall†	9/964 (0.93 [0.43–1.76])	2/1,079 (0.19 [0.02–0.67])	4/1,545 (0.26 [0.07–0.66])	0.03
H9N2				
2013	1/511 (0.20 [0.01–1.09])	0/569 (0 [0–0.65])	2/915 (0.22 [0.03–0.79])	0.61
2014	2/533 (0.38 [0.05–1.35])	1/589 (0.17 [0–0.94])	1/881 (0.11 [0–0.63])	0.70
2015	11/535 (2.06 [1.03–3.65])	0/501 (0 [0–0.73])	4/855 (0.47 [0.13–1.19])	<0.001
2016	7/491 (1.43 [0.58–2.92])	3/367 (0.82 [0.17–2.37])	2/785 (0.25 [0.03–0.92])	0.05
Overall†	18/964 (1.87 [1.11–2.94])	3/1,079 (0.28 [0.06–0.81])	9/1,545 (0.58 [0.27–1.10])	<0.001
H5N1				
2013	1/511 (0.20 [0–1.09])	0/569 (0 [0–0.65])	0/915 (0 [0–0.40])	0.26
2014	0/533 (0 [0–0.69])	0/589 (0 [0–0.62])	0/881 (0 [0–0.42])	NA
2015	0/535 (0 [0–0.69])	0/501 (0 [0–0.73])	0/855 (0 [0–0.43])	NA
2016	17/491 (3.46 [2.03–5.49])	0/367 (0 [0–1.00])	0/785 (0 [0–0.47])	<0.001
Overall†	18/964 (1.87 [1.11–2.94])	0/1,079 (0 [0–0.34])	0/1,545 (0 [0–0.24])	<0.001

*NA, the statistics were not performed because of 0 in the 2 groups. †The overall seroprevalence was calculated as the number of seropositive persons divided by the number of all new enrolled persons during the study period.

### Seroprevalence

Seroprevalence differed by group and over time (Table 1). The overall seroprevalence of H7N9, H9N2, and H5N1 viruses in poultry workers was significantly higher than in swine workers and controls (p<0.05). Of 964 enrolled poultry workers, 9 (0.93% [95% CI 0.43%–1.76%]) were seropositive for H7N9, 18 (1.87% [95% CI 1.11%–2.94%]) for H9N2, and 18 (1.87% [95% CI 1.11%–2.9%]) for H5N1 during the study period. In comparison, of 1,079 enrolled swine workers, only 2 (0.19% [95% CI 0.02%–0.67%]) were seropositive for H7N9 and 3 (0.28% [95% CI 0.06%–0.81%]) for H9N2. Similar seroprevalence was observed among the 1,545 enrolled controls. No poultry workers were found seropositive for H7N9 in the 2016 survey and for H5N1 virus in the 2014 and 2015 surveys. In addition, we observed a significant increase in seroprevalence of 3.46% for H5N1 virus among poultry workers in the 2016 survey, compared with the previous year’s survey. No participants in any group were seropositive for H5N6 throughout the study.

### Incidence of Seroconversion

During the study period, 30 participants seroconverted (Table 2). Among the poultry workers, 2 (0.43%) seroconverted for H7N9, 13 (2.78%) for H9N2, and 7 (1.5%) for H5N1 (Table 3), resulting in incidences of 1.27/1,000 person-years for H7N9, 8.28/1,000 person-years for H9N2, and 4.46/1,000 person-years for H5N1 (Table 4). Among swine workers and controls, only 1 control seroconverted for the H7N9 virus, and 3 (0.58%) swine workers and 4 (0.39%) controls seroconverted for H9N2 (Table 3). Although the incidence among swine workers and controls was low or 0 for H7N9 and H5N1, the incidence of H9N2 was relatively high among swine workers (1.93/1,000 person-years) and controls (1.54/1,000 person-years) (Table 4). Poultry workers were more likely than controls to have infection with H9N2 (incidence rate ratio 5.36 [95% CI 1.65%–22.55%]) and H5N1, but seroconversion rates between the groups did not differ significantly for H7N9 (Table 4).

**Table 2:** Characteristics of poultry workers, swine workers, and controls with seroconversion of influenza A(H7N9), A(H9N2), and A(H5N1) viruses, eastern China, 2013–2016*

Virus, participant no.	Age, y/sex	Occupation	Chronic medical condition	MN titer			
2013	2014	2015	2016
H7N9							
1	28/F	Chicken slaughtering	No	40	320	5	5
2	41/F	Chicken slaughtering	No	5	5	320	NA
3	63/F	Retired	No	20	80	NA	NA
H9N2							
4	48/F	Chicken backyard grower	No	5	5	80	40
5	28/M	Chicken raising	No	NA	5	80	80
6	51/F	Chicken raising	No	5	5	5	80
7	47/F	Chicken seller	No	5	20	80	40
8	47/M	Chicken seller	No	5	5	160	NA
9	46/M	Chicken seller	No	5	5	160	NA
10	51/M	Chicken seller	Chronic bronchitis	5	40	160	NA
11	49/M	Chicken/duck seller	Diabetes	NA	NA	20	80
12	59/F	Chicken/duck seller	No	5	5	80	320
13	39/F	Chicken/duck seller	No	5	NA	20	80
14	27/F	Chicken/goose seller	No	5	320	40	40
15	57/F	Chicken/pigeon slaughtering	No	5	40	80	5
16	52/F	Duck/goose seller	No	5	80	5	5
17	32/M	Pig slaughtering	No	5	5	5	80
18	52/M	Pig slaughtering	No	5	80	NA	5
19	26/M	Pork seller	No	5	5	5	160
20	40/M	Grocer, control	Chronic bronchitis	5	160	5	5
21	48/M	Grocer, control	No	5	5	80	5
22	38/M	Grocer, control	Diabetes	5	5	160	5
23	61/M	Retired, control	No	NA	5	5	80
H5N1							
24	39/F	Chicken/duck/goose seller	No	5	5	20	80
25	45/F	Chicken/duck/pigeon raising	No	20	10	40	80
26	48/M	Pigeon seller	No	10	10	10	80
27	60/F	Chicken/goose seller	No	10	5	40	80
28	55/F	Duck/goose seller	No	5	5	40	160
29	46/F	Chicken slaughtering	No	40	20	20	80
30	53/F	Chicken slaughtering	No	20	5	20	80

*MN, microneutralization; NA, the participant was not available in this year.

**Table 3:** Seroconversion of microneutralization titers against influenza A(H7N9), A(H9N2), and A(H5N1) viruses in poultry workers, swine workers, and controls, eastern China, 2013–2016

Virus	No. seropositive/no. total (% [95% CI])		
Poultry workers	Swine workers	Controls
H7N9	2/468 (0.43 [0.05–1.54])	0/514 (0 [0–0.72])	1/1030 (0.10 [0.00–0.54])
H9N2	13/468 (2.78 [1.48–4.70])	3/514 (0.58 [0.12–1.70])	4/1030 (0.39 [0.11–0.99])
H5N1	7/468 (1.50 [0.60–3.06])	0/514 (0 [0–0.72])	0/1030 (0 [0–0.36])

**Table 4:** Seroincidence of influenza A(H7N9), A(H9N2), and A(H5N1) viruses in in poultry workers, swine workers, and controls, eastern China, 2013–2016*

Antigen, participant category	Person-years	No. seroconversions	Incidence (95% CI)	IRR (95% CI)
H7N9				
Poultry workers	1,569	2	1.27 (0.15–4.60)	3.30 (0.17–194.48)
Swine workers	1,558	0	0 (0–2.36)	1.66 (0–64.73)
Controls	2,586	1	0.39 (0.01–2.15)	Reference
H9N2				
Poultry workers	1,569	13	8.28 (4.42–14.12)	5.36 (1.65–22.55)
Swine workers	1,558	3	1.93 (0.40–5.61)	1.24 (0.18–7.36)
Controls	2,586	4	1.54 (0.42–3.96)	Reference
H5N1				
Poultry workers	1,569	7	4.46 (1.80–9.17)	NA
Swine workers	1,558	0	0 (0–2.36)	NA
Controls	2,586	0	0 (0–1.43)	Reference

*Incidence is per 1,000 person-years. IRR, incidence rate ratio; NA, statistics not performed because of 0 in 2 groups.

### Risk Factors for AIV Infections

Poultry workers who performed selling had 4.25 (95% CI 1.20–25.32) times higher odds of H9N2 virus infection than did poultry workers who performed slaughtering (Table 5). Among poultry workers, female sex (adjusted OR 5.48 [95% CI 2.38–12.62]) and exposure to pigeons (adjusted OR 3.13 [95% CI 1.23–8.00]) were also significant risk factors for H5N1 virus seropositivity or seroconversion. Controls who were male (adjusted OR 8.75 [95% CI 1.09–70.45]) or had chronic respiratory disease (adjusted OR 7.24 [95% CI 1.42–37.00]) were more likely to be seropositive or to seroconvert for H9N2.

**Table 5:** Risk factors for testing seropositive or seroconverting against influenza A(H9N2) and A(H5N1) viruses among poultry workers and controls, eastern China, 2013–2016*

Risk factor, antigen	Total	Seropositive or seroconverted, no. (%)	Crude OR (95% CI)	Adjusted OR (95% CI)
Poultry worker				
H9N2	468	18 (3.85)		
Exposure behavior†				
Selling	181	13 (7.18)	4.68 (1.04–21.13)	4.25 (1.20–25.32)
Raising	198	3 (1.52)	0.93 (0.15–5.65)	1.12 (0.18–6.85)
Cleaning	134	1 (0.75)	0.46 (0.04–5.08)	0.22 (0.05–4.99)
Transporting	46	0	NA	NA
Slaughtering	123	2 (1.63)	Reference	Reference
H5N1	468	18 (3.85)		
Sex				
F	222	14 (6.31)	4.07 (1.32–12.56)	5.48 (2.38–12.62)
M	246	4 (1.63)	Reference	Reference
Exposure†				
Goose	49	5 (10.20)	3.24 (1.11–9.42)	2.64 (0.72–9.74)
Pigeon	66	6 (9.09)	2.85 (1.06–7.70)	3.13 (1.23–8.00)
Duck	104	7 (6.73)	2.06 (0.81–5.23)	1.87 (0.77–5,01)
Chicken	413	14 (3.39)	Reference	Reference
Controls, H9N2	1030	9 (0.87)		
Sex				
M	495	8 (1.62)	8.77 (1.10–70.39)	8.75 (1.09–70.45)
F	535	1 (0.19)	Reference	Reference
Chronic respiratory disease				
Yes	38	2 (5.26)	7.82 (1.57–38.96)	7.24 (1.42–37.00)
No	992	7 (0.71)	Reference	Reference

*OR, odds ratio; NA, statistics not performed because of 0 in 2 or 3 groups. †Participants might be included in multiple categories.

### Diversity and Reassortment of AIVs at LPMs

**Figure 3:**
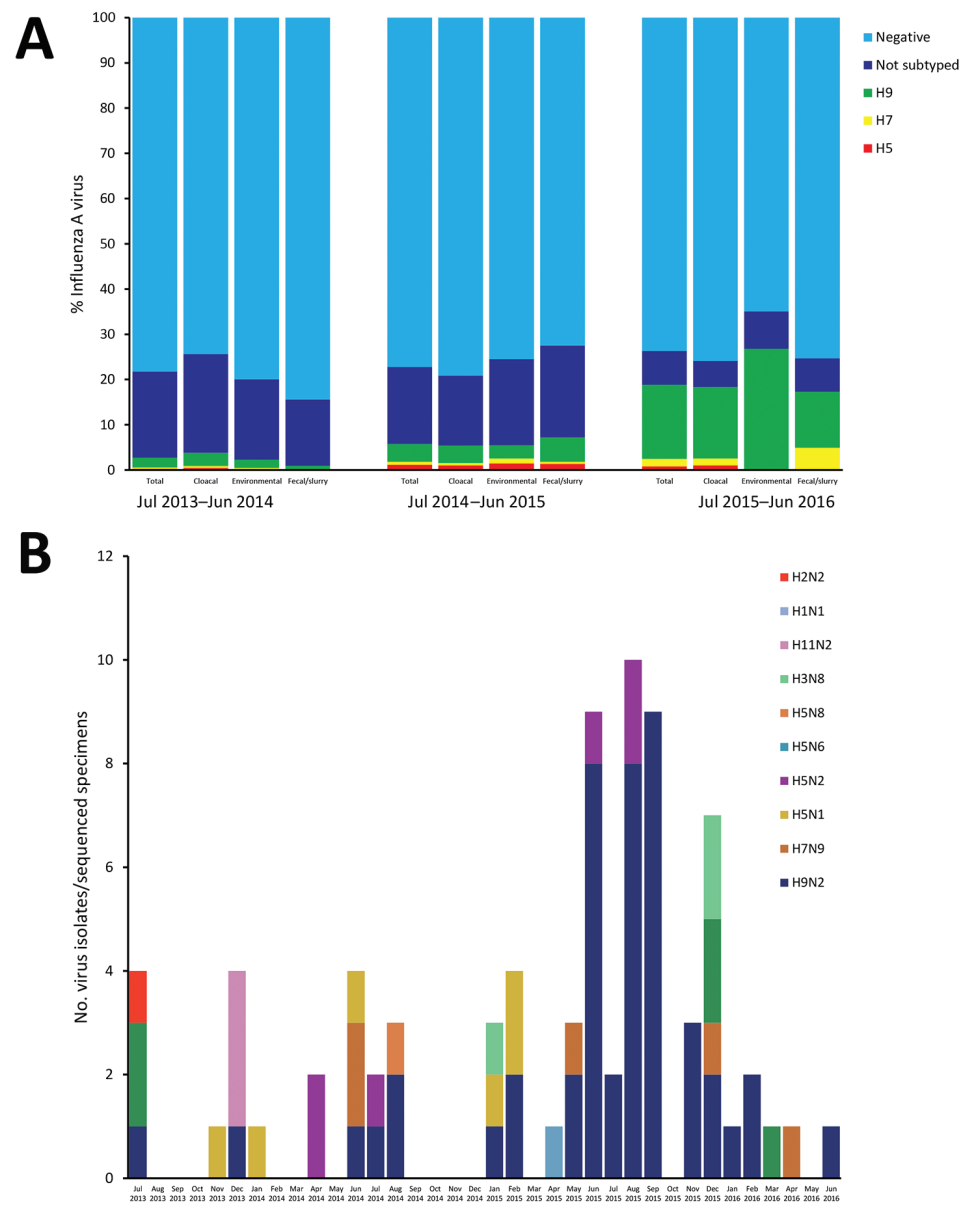
Influenza A virus detection in samples from live poultry markets, Wuxi, Jiangsu Province, China, 2013–2016. A) Proportion of H9, H7, and H5 subtype detection in cloacal swab, environmental swabs, and fecal/slurry samples; B) genetic classification and number of influenza isolates and sequenced specimens over time. Some could not be subtyped because of weakly positive laboratory results.

**Figure 4:**
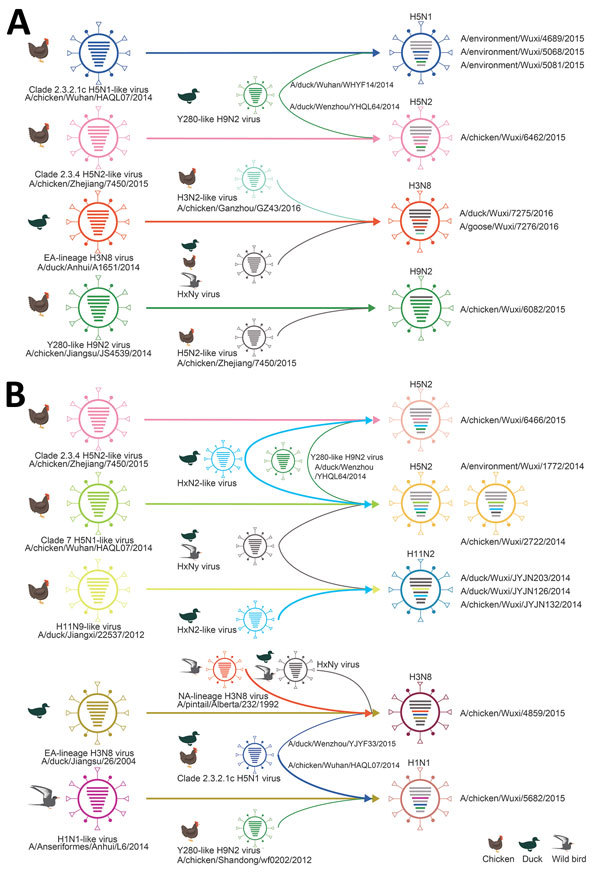
Probable genesis of reassortant influenza A viruses, Wuxi, Jiangsu Province, China, 2013–2016. A) Internal gene reassortment; B) hemagglutinin, neuraminidase, and internal gene exchanges. Virus particles are represented by ovals containing horizontal bars that represent the 8 gene segments (top to bottom: polymerase basic 2, polymerase basic 1, polymerase acidic, hemagglutinin, nucleoprotein, neuraminidase, matrix, and nonstructural); colors indicate sequence origin based on initial viruses shown at far left (gray bars indicate no sequence data available).

During the study period, we collected and screened 3,121 samples from 9 LPMs for IAVs. A total of 466 (23.2%) of 2,010 cloacal swab samples, 145 (24.5%) of 590 environmental swab samples, and 115 (22.0%) of 521 fecal/slurry specimens were positive for influenza A (Figure 3, panel A). Single infection with H9, H7, and H5 subtypes was detected in 229 (31.5%), 27 (3.7%), and 25 (3.4%) of 726 AIV-positive specimens, respectively. Sequencing results of 45 isolated strains and 33 original specimens (Technical Appendix Table 3) showed that 10 AIV subtypes were detected in LPMs (Figure 3, panel B). To further study the origin of these 10 subtypes, we performed a detailed phylogenetic analysis for all available gene segments (Technical Appendix Figure). The analyses revealed multiple gene segment exchanges among and within subtypes or interspecies among those circulating in domestic and wild birds, resulting in new genetic constellations and reassortant viruses, which we have represented schematically (Figure 4). Overall, 2 reassortment models were observed for these viruses. The 1 reassortment model mentioned only internal gene reassortment (Figure 4, panel A), such as the matrix (M) gene of 3 H5N1 (A/environment/Wuxi/4689/2015, A/environment/Wuxi/5068/2015, and A/environment/Wuxi/5081/2015) and 1 H5N2 (A/chicken/Wuxi/6462/2015) virus originating from Y280-like H9N2 viruses, the polymerase basic 2 gene of 1 H9N2 (A/chicken/Wuxi/6082/2015) virus from A/chicken/Zhejiang/7450/2015 H5N2-like virus, and all internal genes of 2 H3N8 (A/duck/Wuxi/7275/2016 and A/goose/Wuxi/7276/2016) viruses multireasserted from chicken or wild bird HxNy–like virus. The other reassortment model included multireassortment involving both the surface protein genes (hemagglutinin [HA], neuraminidase [NA], or both) and internal genes among the different subtypes or lineage and interspecies (Figure 4, panel B). The HA gene of 3 H5N2 viruses originated from clade 2.3.4 H5N2 (A/chicken/Wuhan/HAQL07/2014) or clade 7 H5N1 (A/chicken/Zhejiang/7450/2015)-like viruses and the HA gene of 3 H11N2 viruses from A/duck/Jiangxi/22537/2012-like H11N9 virus, the NA gene in all of them was originated from HxN2-like viruses. The 1 H3N8 virus (A/chicken/Wuxi/4859/2015) also had multireassortments that the HA and NA were respectively generated from the HA of Eurasian (A/duck/Jiangsu/26/2004) and North American lineage (A/pintail/Alberta/232/1992) H3N8-like virus, and the internal genes were reassorted with 6 subtypes circulating in ducks and wild birds. The HA of H1N2-like virus (A/Anseriformes/Anhui/L6/2014), the NA of clade 2.3.2.1c H5N1-like virus (A/chicken/Wuhan/HAQL07/2014), and the M gene of Y-280 lineage H9N2-like virus (A/chicken/Shandong/wf0202/2012) reassorted and generated new H1N1 virus (A/chicken/Wuxi/5682/2015).

All H5 subtypes possessed a polybasic amino acid residue at the cleavage site (RERRRKR/GL), indicating they were highly pathogenic in chickens, whereas the other subtypes were low pathogenicity (Technical Appendix Table 3). We detected the HA Q226L (H3 numbering) mutation in 4 H7N9 and all H9N2 viruses, indicating a binding ability to the human-like receptor. However, all subtypes had no polymerase basic 2 E627K and D701N mutations. All H7, H9, and H5 subtypes had the deletion in NA stalk associated with enhanced virulence in mice, as well as adaptation and transmission in poultry. All H9N2 viruses had oseltamivir resistance mutations of R292K in NA (N2 numbering), and adamantine resistance-associated mutation of S31N of M2 protein in 2 H5N1 and all H5N2, H1N1, H7N9, and H9N2 viruses.

## Discussion

We estimated the seroprevalence and seroincidence of H7N9, H9N2, H5N1, and H5N6 viruses in an open cohort of poultry workers, swine workers, and the general population in Wuxi, Jiangsu Province, China. Poultry workers had relatively higher seroprevalence and seroincidence of H7N9, H9N2, and H5N1 than swine workers and the general population, although the overall seroprevalence and seroincidence was low. Active surveillance for AIVs revealed that 10 subtypes were circulating at LPMs, and extensive gene segment reassorts occurred among and within subtype or interspecies that circulate in domestic poultry and wild birds.

Serologic evidence of human infection with H7N9 has previously been reported (*14*–*18*,*22*–*24*). In those studies, the seroprevalence ranged from 0% to 17.1%. In our study, a much lower seroprevalence of anti-H7N9 virus ranged from 0% to 0.56% during the enrollment and follow-up times in poultry workers. Our findings are similar to the 0.11% seroprevalence of MN titers >20 found in poultry workers in 6 provinces in China (*18*). In comparison with studies that did not perform MN testing, the proportion of elevated HI titers >20 in our study was also much lower (0%–2.83% in poultry workers). For example, 7.2%–14.9% of poultry workers in Shenzhen had HI titers >160 (*14*). Another study found that 1.6% of poultry workers with HI titers >40 in Guangzhou (*22*); 2 studies in Zhejiang Province reported that 3.7% and 6.3% of poultry workers had HI titers >80 (*16*,*24*); a study in Taiwan reported 0% of poultry workers with HI titers >10 (*23*). In our study, swine workers and general population controls had an extremely low seroprevalence of the H7N9 virus, similar to the results of serologic studies in southern China (*14*–*16*). Our observed low seroprevalence is not surprising because the number of reported H7N9 cases and potential H7N9-positive markets in Wuxi was small during the study period. Differences in seroprevalence across studies also could be explained by differences between serologic assays because different tests might have marked sensitivity/specificity and high interstudy variability. Although the findings from our study and these early serologic studies reassuringly suggest that the number of undetected cases of H7N9 virus was low, close monitoring of transmission remains essential as the virus and epidemic continued to evolve.

Human infections with H9N2 virus have been reported since 1998, and concern about its pandemic potential has increased, especially in recent years. Because this virus always causes mild upper respiratory tract illness that is clinically indistinguishable from the symptoms of common influenza caused by seasonal human H1N1 and H3N2 viruses, the incidence of H9N2 infections might be underestimated. Previous studies in China (*25*–*28*) and other countries (*29*–*35*) estimated that seroprevalence ranged from 0.5% to 4.6% in poultry workers. Our results showed that poultry workers had an overall H9N2 seroprevalence of 1.87% and a seroincidence of 8.78/1,000 person-years, which is significantly higher than those of H7N9 and H5N1. We detected no significant serologic response at baseline, but the seropositive rate increased considerably during the next 3 follow-up points in poultry workers. This finding seems to be consistent with an increased prevalence (2.73% during July 2013–June 2014, 5.10% during July 2014–June 2015, and 22.22% during July 2015–June 2016) of H9N2 viruses detected in poultry at LPMs.

We also tested clade 2.3.2.1c H5N1 and clade 2.3.4.4 H5N6 viruses. Overall, the seroprevalence of H5N1 was low, and only poultry workers in 2016 had seropositive titers for a seroprevalence of 3.46%, which was similar to findings from studies conducted in southern China during the same period (*14*,*36*,*37*). Also, antibody levels were relatively low (the highest titer was 160), consistent with the low immunogenicity of H5N1 (*38*). Since the first H5N6 infections in humans reported in China in 2014, a total of 17 cases have been reported, but none of the participants in our study were seropositive or seroconverted during the study period. However, the circulation of H5N6 in LPMs and the continuous reassortment of their internal genes with Y280-like H9N2 virus remains a potential cause of human infections.

Our active surveillance data revealed a high diversity of AIVs at LPMs. We observed genetic evidence of extensive reassortment of viral genes among and within subtype, and the new viral genes were introduced from the wild bird gene pool to domestic poultry, which further enriched such diversity. Additional co-detections of H9N2 with H7N9, H5N1, or H5N6 might provide the potential conditions for intersubtype reassortment. Our data also showed that H9N2 was the dominant circulating subtype, showing a high prevalence of 31.5%. Furthermore, all or some of the internal genes of the viruses we identified were from the Y280-like H9N2 virus, such as H7N9, H5N1, H5N2, and H1N1. All H5 subtype viruses showed a polybasic cleavage site, indicating its high pathogenicity in poultry. Although no outbreaks of H5 subtype viruses were reported in Wuxi, the outbreaks of H5N1, H5N2, and H5N6 in poultry were reported in several cities of Jiangsu Province surrounding Wuxi (*39*). Because H9N2 is not highly pathogenic, the extent of infection in poultry and humans is likely to remain underappreciated. Interaction or reassortment between the prevailing human and avian influenza viruses is considered the most probable scenario for generating new pandemic strains. We also argue that almost anywhere in the world where LPMs exist, especially large LPMs with different poultry, disparate viruses could be mixed yielding new AIVs. These viruses can move quickly across large geographic areas and change rapidly. Hence, our findings support the conclusion that LPMs play a critical role in the continual emergence of new reassortant AIVs that can spread through poultry populations. Thus, influenza surveillance among wild bird and domestic poultry at LPMs should be strengthened.

Our study had several limitations. First, although our study provides serologic evidence of virus infection, we did not conduct surveillance for influenza-like illness among participants, which prevents us from identifying laboratory-confirmed human disease and obtaining evidence of direct transmission from poultry to humans. Second, because of possible waning of antibodies or lack of antibody response to AIVs during the 1-year follow-up period, we were unlikely to have detected all seroconversions during the study period; thus, our study might underestimate the seroincidence.

In conclusion, conducting surveillance for new influenza virus surveillance at LPMs, especially when the LPMs are large and can sustain virus transmission, and monitoring the poultry and poultry workers for the new AIV infections are critical. Despite overall low seroprevalence or seroincidence, poultry workers had a higher risk for infection than swine workers and controls. Thus, it seems prudent to encourage poultry workers to use personal protective equipment (e.g., masks and gloves) and to undergo educational programs to help them understand and prevent AIV transmission between humans and poultry.

Technical Appendix Additional methods and results, characteristics of study participant, and characteristics of molecular markers of the influenza A viruses studied.

## References

[1] Gao R, Cao B, Hu Y, Feng Z, Wang D, Hu W, et al. Human infection with a novel avian-origin influenza A (H7N9) virus. N Engl J Med. 2013;368:1888–97. 10.1056/NEJMoa1304459 23577628

[2] Pan M, Gao R, Lv Q, Huang S, Zhou Z, Yang L, et al. Human infection with a novel, highly pathogenic avian influenza A (H5N6) virus: Virological and clinical findings. J Infect. 2016;72:52–9. 10.1016/j.jinf.2015.06.009 26143617

[3] Chen H, Yuan H, Gao R, Zhang J, Wang D, Xiong Y, et al. Clinical and epidemiological characteristics of a fatal case of avian influenza A H10N8 virus infection: a descriptive study. Lancet. 2014;383:714–21. 10.1016/S0140-6736(14)60111-2 24507376

[4] Flutracker. China H9N2, H5N1, H5N8, H5N6, H5N3, H5N2, H10N8 outbreak tracking [cited 2017 May 27]. https://flutrackers.com/forum/

[5] Zhang L, Zhang Z, Weng Z, Shi W. Substitution rates of the internal genes in the novel avian H7N9 influenza virus. Clin Infect Dis. 2013;57:1213–5. 10.1093/cid/cit447 23821733

[6] Jiang H, Wu P, Uyeki TM, He J, Deng Z, Xu W, et al. Preliminary epidemiologic assessment of human infections with highly pathogenic avian influenza A(H5N6) virus, China. Clin Infect Dis. 2017;65:383–8. 10.1093/cid/cix334 28407105 PMC5848334

[7] Ip DK, Liao Q, Wu P, Gao Z, Cao B, Feng L, et al. Detection of mild to moderate influenza A/H7N9 infection by China’s national sentinel surveillance system for influenza-like illness: case series. BMJ. 2013;346(jun24 1):f3693. 10.1136/bmj.f3693 23798720 PMC3691004

[8] Xu C, Havers F, Wang L, Chen T, Shi J, Wang D, et al. Monitoring avian influenza A(H7N9) virus through national influenza-like illness surveillance, China. Emerg Infect Dis. 2013;19:1289–92. 10.3201/eid1907.130662 23879887 PMC3739526

[9] Yang P, Pang X, Deng Y, Ma C, Zhang D, Sun Y, et al. Surveillance for avian influenza A(H7N9), Beijing, China, 2013. Emerg Infect Dis. 2013;19:2041–3. 10.3201/eid1912.130983 24274700 PMC3840857

[10] Chakraborty A, Rahman M, Hossain MJ, Khan SU, Haider MS, Sultana R, et al. Mild respiratory illness among young children caused by highly pathogenic avian influenza A (H5N1) virus infection in Dhaka, Bangladesh, 2011. J Infect Dis. 2017;216(suppl_4):S520–S8. 10.1093/infdis/jix019 PMC11753186 28934459

[11] Yuan R, Liang L, Wu J, Kang Y, Song Y, Zou L, et al. Human infection with an avian influenza A/H9N2 virus in Guangdong in 2016. J Infect. 2017;74:422–5. 10.1016/j.jinf.2017.01.003 28109675

[12] Khan SU, Anderson BD, Heil GL, Liang S, Gray GC. A systematic review and meta-analysis of the seroprevalence of influenza A(H9N2) infection among humans. J Infect Dis. 2015;212:562–9. 10.1093/infdis/jiv109 25712969 PMC4598807

[13] Toner ES, Adalja AA, Nuzzo JB, Inglesby TV, Henderson DA, Burke DS. Assessment of serosurveys for H5N1. Clin Infect Dis. 2013;56:1206–12. 10.1093/cid/cit047 23386633 PMC3858121

[14] Wang X, Fang S, Lu X, Xu C, Cowling BJ, Tang X, et al. Seroprevalence to avian influenza A(H7N9) virus among poultry workers and the general population in southern China: a longitudinal study. Clin Infect Dis. 2014;59:e76–83. 10.1093/cid/ciu399 24867786 PMC4155446

[15] Yang P, Ma C, Cui S, Zhang D, Shi W, Pan Y, et al. Avian influenza A(H7N9) and (H5N1) infections among poultry and swine workers and the general population in Beijing, China, 2013-2015. Sci Rep. 2016;6:33877. 10.1038/srep33877 27670286 PMC5037362

[16] Yang S, Chen Y, Cui D, Yao H, Lou J, Huo Z, et al. Avian-origin influenza A(H7N9) infection in influenza A(H7N9)-affected areas of China: a serological study. J Infect Dis. 2014;209:265–9. 10.1093/infdis/jit430 23935201

[17] Chen J, Ma J, White SK, Cao Z, Zhen Y, He S, et al. Live poultry market workers are susceptible to both avian and swine influenza viruses, Guangdong Province, China. Vet Microbiol. 2015;181:230–5. 10.1016/j.vetmic.2015.09.016 26476563 PMC7119354

[18] Xiang N, Bai T, Kang K, Yuan H, Zhou S, Ren R, et al. Sero-epidemiologic study of influenza A(H7N9) infection among exposed populations, China 2013-2014. Influenza Other Respi Viruses. 2017;11:170–6. 10.1111/irv.12435 27762061 PMC5304573

[19] Fuller TL, Gilbert M, Martin V, Cappelle J, Hosseini P, Njabo KY, et al. Predicting hotspots for influenza virus reassortment. Emerg Infect Dis. 2013;19:581–8. 10.3201/eid1904.120903 23628436 PMC3647410

[20] World Health Organization. Manual for the laboratory diagnosis and virological surveillance of influenza. 2011 [cited 2011 Dec 10]. http://apps.who.int/iris/bitstream/10665/44518/1/9789241548090_eng.pdf

[21] Rowe T, Abernathy RA, Hu-Primmer J, Thompson WW, Lu X, Lim W, et al. Detection of antibody to avian influenza A (H5N1) virus in human serum by using a combination of serologic assays. J Clin Microbiol. 1999;37:937–43. 10074505 10.1128/jcm.37.4.937-943.1999 PMC88628

[22] Chen Z, Li K, Luo L, Lu E, Yuan J, Liu H, et al. Detection of avian influenza A(H7N9) virus from live poultry markets in Guangzhou, China: a surveillance report. PLoS One. 2014;9:e107266. 10.1371/journal.pone.0107266 25216284 PMC4162608

[23] Huang SY, Yang JR, Lin YJ, Yang CH, Cheng MC, Liu MT, et al. Serological comparison of antibodies to avian influenza viruses, subtypes H5N2, H6N1, H7N3 and H7N9 between poultry workers and non-poultry workers in Taiwan in 2012. Epidemiol Infect. 2015;143:2965–74. 10.1017/S0950268815000394 25761403 PMC9151060

[24] He F, Chen EF, Li FD, Wang XY, Wang XX, Lin JF. Human infection and environmental contamination with Avian Influenza A (H7N9) Virus in Zhejiang Province, China: risk trend across the three waves of infection. BMC Public Health. 2015;15:931. 10.1186/s12889-015-2278-0 26392274 PMC4576372

[25] Zhou P, Zhu W, Gu H, Fu X, Wang L, Zheng Y, et al. Avian influenza H9N2 seroprevalence among swine farm residents in China. J Med Virol. 2014;86:597–600. 10.1002/jmv.23869 24390939

[26] Huang R, Wang AR, Liu ZH, Liang W, Li XX, Tang YJ, et al. Seroprevalence of avian influenza H9N2 among poultry workers in Shandong Province, China. Eur J Clin Microbiol Infect Dis. 2013;32:1347–51. 10.1007/s10096-013-1888-7 23733318

[27] Wang M, Fu CX, Zheng BJ. Antibodies against H5 and H9 avian influenza among poultry workers in China. N Engl J Med. 2009;360:2583–4. 10.1056/NEJMc0900358 19516044

[28] Yu Q, Liu L, Pu J, Zhao J, Sun Y, Shen G, et al. Risk perceptions for avian influenza virus infection among poultry workers, China. Emerg Infect Dis. 2013;19:313–6. 10.3201/eid1902.120251 23343592 PMC3563274

[29] Gray GC, McCarthy T, Capuano AW, Setterquist SF, Alavanja MC, Lynch CF. Evidence for avian influenza A infections among Iowa’s agricultural workers. Influenza Other Respi Viruses. 2008;2:61–9. 10.1111/j.1750-2659.2008.00041.x 18941621 PMC2568886

[30] Gomaa MR, Kayed AS, Elabd MA, Zeid DA, Zaki SA, El Rifay AS, et al. Avian influenza A(H5N1) and A(H9N2) seroprevalence and risk factors for infection among Egyptians: a prospective, controlled seroepidemiological study. J Infect Dis. 2015;211:1399–407. 10.1093/infdis/jiu529 25355942 PMC4462653

[31] Ahad A, Thornton RN, Rabbani M, Yaqub T, Younus M, Muhammad K, et al. Risk factors for H7 and H9 infection in commercial poultry farm workers in provinces within Pakistan. Prev Vet Med. 2014;117:610–4. 10.1016/j.prevetmed.2014.10.007 25457514

[32] Coman A, Maftei DN, Krueger WS, Heil GL, Friary JA, Chereches RM, et al. Serological evidence for avian H9N2 influenza virus infections among Romanian agriculture workers. J Infect Public Health. 2013;6:438–47. 10.1016/j.jiph.2013.05.003 23999337

[33] Blair PJ, Putnam SD, Krueger WS, Chum C, Wierzba TF, Heil GL, et al. Evidence for avian H9N2 influenza virus infections among rural villagers in Cambodia. J Infect Public Health. 2013;6:69–79. 10.1016/j.jiph.2012.11.005 23537819 PMC3612269

[34] Uyeki TM, Nguyen DC, Rowe T, Lu X, Hu-Primmer J, Huynh LP, et al. Seroprevalence of antibodies to avian influenza A (H5) and A (H9) viruses among market poultry workers, Hanoi, Vietnam, 2001. PLoS One. 2012;7:e43948. 10.1371/journal.pone.0043948 22928049 PMC3424239

[35] Pawar SD, Tandale BV, Raut CG, Parkhi SS, Barde TD, Gurav YK, et al. Avian influenza H9N2 seroprevalence among poultry workers in Pune, India, 2010. PLoS One. 2012;7:e36374. 10.1371/journal.pone.0036374 22623954 PMC3356154

[36] Matrosovich MN, Krauss S, Webster RG. H9N2 influenza A viruses from poultry in Asia have human virus-like receptor specificity. Virology. 2001;281:156–62. 10.1006/viro.2000.0799 11277689

[37] Yang P, Ma C, Shi W, Cui S, Lu G, Peng X, et al. A serological survey of antibodies to H5, H7 and H9 avian influenza viruses amongst the duck-related workers in Beijing, China. PLoS One. 2012;7:e50770. 10.1371/journal.pone.0050770 23226380 PMC3511333

[38] Clegg CH, Roque R, Van Hoeven N, Perrone L, Baldwin SL, Rininger JA, et al. Adjuvant solution for pandemic influenza vaccine production. Proc Natl Acad Sci U S A. 2012;109:17585–90. 10.1073/pnas.1207308109 23045649 PMC3491477

[39] World Organization of Animal Health. Latest updates on avian Influenza. 2018 [cited 2018 Mar 9]. http://www.oie.int/en/animal-health-in-the-world/update-on-avian-influenza

