# Supplementary material for: Avian Influenza A Virus Infection among Workers at Live Poultry Markets, China, 2013–2016
Source: Emerg Infect Dis. 2018 Jul;24(7):1246–56. doi: 10.3201/eid2407.172059 (PMC6038753; doi:10.3201/eid2407.172059)
Supplement: Technical Appendix — Additional methods and results, characteristics of study participant, and characteristics of molecular markers of the influenza A viruses studied. [file 17-2059-Techapp-s1.pdf]

Article DOI: https://doi.org/10.3201/eid2407.172059

Avian Influenza A Virus Infection among Workers at Live Poultry Markets, China, 2013–2016

Technical Appendix

Methods

Serologic Assays

Hemagglutination-inhibition (HI) assay was performed as previously described (*1*). Briefly, after treatment by 1:4 solution of receptor-destroying enzyme (Denka Seiken Co Ltd, Tokyo, Japan) at 37°C for 18 h and then heat-inactivated at 56°C for 30 min to eliminate nonspecific inhibitors, 2-fold serial dilutions of serum (from 1:10 to 1:1,280) were tested against 8 hemagglutinin (HA) units of antigen using 1% horse red blood cells. For the H9N2 virus, 0.5% turkey red cells were used as the receptor of H9N2 virus circulation in China has changed to the human-like receptor. The HI titers were defined as the reciprocal of the highest serum dilution that completely inhibited hemagglutination.

Serum with HI titer $\geq$1:10 were further confirmed by microneutralization (MN) assay as previous described (*2*) using a culture of MDCK cells as with minor adaption. In brief, 2-fold serial dilutions of serum from 1:10 to 1:1,280 were incubated with 100 median 50% tissue culture infective dose of the H7N9 virus. 100 μL MDCK cells suspension with 2.0×10^5 cells/mL to each well and the plate incubated at 37°C with 5% CO_2 for 24 h, followed by ELISA to determine virus titer. The MN titer was expressed as the reciprocal of the highest dilution of serum with optical density (OD) <X, where X = [(average OD of virus control wells) + (average OD of cell control wells)]/2. The MN titer as the reciprocal of the highest serum dilution that yielded >50% neutralization. For final titers <1:10 we assigned a value of 1:5 (seronegative).

Molecular Detection, Viral Isolation, and Sequencing

The viral RNA of each sample was extracted (Cat. No. 9766, TaKaRa, Dalian, China) and was subsequently screened for influenza virus A virus (IAV) by real-time reverse

transcription PCR (rRT-PCR) (cat. no. 56046, TaKaRa, Dalian, China) targeting the influenza matrix genome segment (*3*). These IAV-positive specimens were further subtyped for avian influenza H5, H7, and H9 as previously described (*3*,*4*). Meanwhile, IAV-positive specimens were inoculated into 9–11-day specific pathogen–free embryonated chicken eggs for virus isolation.

The full genome of cultured isolates was amplified (cat: 055A, TaKaRa, Dalian, China) using a pair of universal primers that amplify full-length viral genome sequences (*5*). PCR amplicons were purified (cat. no. 28004, QIAGEN) and then sequenced on Ion Torrent Personal Genome Machine (PGM, Life Technologies, South San Francisco, CA, USA). For samples that failed for virus isolation, HA, neuraminidase (NA), and matrix (M) genes were tried to amplify using universal primers (*6*) for sequencing. The sequence data were deposited in Global Initiative on Sharing All Influenza Data (GISAID) (accession no. EPI_ISL_277027–277050, EPI_ISL_277052–277064, and EPI_ISL_277093–277127).

Phylogenetic Analysis

To understand the molecular epidemiology of identified viruses in the study, we first examined ≥100 closely related sequences for each gene in GenBank and GISAID to infer the overall topology, and then we removed a few extreme outlying sequences from the trees. Maximum-likelihood phylogenetic trees were inferred for available gene segments by using MEGA software, version 6.06 (http://www.megasoftware.net). To assess the robustness of individual nodes on phylogenetic trees, we used a bootstrap resampling process (1,000 replications), the neighbor-joining method, and a best-fit, general time-reversible model of nucleotide substitution.

Results

During July 2013–June 2016, active surveillance of IAVs at 9 LPMs was conducted in Wuxi City, Jiangsu Province, China. A total of 3,121 samples (including 2,010 cloacal swabs, 590 environment swabs, and 521 fecal/slurry samples) were collected. A total of 726 (23.3%) samples, including 466 (23.2%) cloacal swabs, 145 (24.5%) environment swabs, and 115 (22.0%) fecal/slurry samples were rRT-PCR positive for IAVs. Of these 726 IAV-positive specimens, 229 (31.5%), 27 (3.7%), and 25 (3.4%) were single infection with H9, H7, and H5

subtype virus, respectively. co-infections of H7 and H9; H5 and H9; and H5, H7, and H9 were detected in 11 (1.5%), 2 (0.3%), and 1 (0.1%) specimens, respectively. A total of 45 samples were successfully isolated, and the whole-genome sequence was sequenced. The HA, NA, and M genes of 33 original specimens that failed for virus isolation were sequenced. The sequencing data revealed 10 subtypes of AIVs in live poultry markets, including H7N9 (n = 5), H9N2 (n = 45), H5N6 (n = 5), H5N1 (n = 6), H5N2 (n = 6), H11N2 (n = 3), H3N8 (n = 3), H1N1 (n = 1), H2N2 (n = 1), and H5N8 (n = 3).

The phylogeny of the H7N9 viruses showed that they all were derived from the Yangtze River Delta lineage associated with the 2013 outbreak of H7N9 in southern China (echnical Appendix Figure, panel A). For the H9N2 viruses, their HA and NA genes were fell into the Y280-like lineage, while internal genes were closely related with those of H9N2viruses in China except for the polymerase basic 2 gene of A/chicken/Wuxi/6082/2015 that was closely related to H5N2 and H5N8 viruses identified in poultry and wild birds, suggestive of reassortment (Technical Appendix Figure, panel B). The 3 H5N6 viruses fell into Jiangxi lineage, which belonged to clade 2.3.4.4, and all gene segments of the H5N6 viruses were closely related to those H5N6 viruses circulating in China and Japan but differed from our previously reported 2 Sichuan lineage–like H5N6 viruses (*7*) (Technical Appendix Figure, panel C). While the 4 H5N1 viruses were similar to our previously reported (*7*) and those of H5N1 viruses circulating in China and Vietnam, the M gene was closely related to those H5N1, and H5N1 containing M gene of Y280-like H9N2 viruses identified in China (Technical Appendix Figure, panel D). The HA genes of 5 newly identified H5N2 viruses fell into clade 2.3.4.4 and clade 7. The NA gene of these viruses was closely related to HxN2 viruses circulating in China and Vietnam, while the M gene was closely related to viruses of Y280-like H9N2 and H5 subtypes circulating in China, suggesting multiple reassortment occurred (Technical Appendix Figure, panel E). The HA gene of the 3 H11N2 viruses was closely related to H11Ny subtype viruses circulating in China and Thailand. Whereas the NA and 6 internal genes were derived from the HxN2 subtypes and wild bird origin subtypes circulating in Asian, respectively, indicating that it was in fact derived from multiple and interspecies reassortment events (Technical Appendix Figure, panel F). The HA gene of all three H3N8 viruses was Eurasian lineage and was closely related to those H3Ny subtypes in China, whereas the NA gene fell into Eurasian and North American lineage. The internal genes of the H3N8 viruses were most likely derived from HxNy subtypes in wild birds,

H3N2 and 2.3.2.1c H5N1 viruses in poultry, suggesting multiple and interspecies reassortment (Technical Appendix Figure, panel G). The HA gene of the H1N1 virus was closely related to H1 from multiple subtypes circulating in wild bird and belonged to the Eurasian avian lineage. However, the NA gene was closely related to clade 2.3.2.1c H5N1 viruses, while the M gene was closely related to H5N1 viruses containing M segment of Y280-like H9N2 viruses circulating in China, suggesting an interspecies reassortment occurred among H1Ny subtypes, H5N1, and H9N2 viruses (Technical Appendix Figure, panel H).

References

1. World Health Organization. Manual for the laboratory diagnosis and virological surveillance of influenza. 2011 [cited 2011 Dec 10]. http://apps.who.int/iris/bitstream/10665/44518/1/9789241548090_eng.pdf

2. Rowe T, Abernathy RA, Hu-Primmer J, Thompson WW, Lu X, Lim W, et al. Detection of antibody to avian influenza A (H5N1) virus in human serum by using a combination of serologic assays. J Clin Microbiol. 1999;37:937–43. PubMed

3. World Health Organization. Real-time RT-PCR protocol for the detection of avian influenza A(H7N9) virus. 15 April 2013 [cited 2017 May 27]. http://www.who.int/influenza/gisrs_laboratory/cnic_realtime_rt_pcr_protocol_a_h7n9.pdf?ua=1

4. World Health Organization. WHO information for molecular diagnosis of influenza virus—update. [cited 2017 May 27]. http://www.who.int/influenza/gisrs_laboratory/molecular_diagnosis/en/

5. Zhou B, Donnelly ME, Scholes DT, St George K, Hatta M, Kawaoka Y, et al. Single-reaction genomic amplification accelerates sequencing and vaccine production for classical and Swine origin human influenza a viruses. J Virol. 2009;83:10309–13. PubMedhttp://dx.doi.org/10.1128/JVI.01109-09

6. Hoffmann E, Stech J, Guan Y, Webster RG, Perez DR. Universal primer set for the full-length amplification of all influenza A viruses. Arch Virol. 2001;146:2275–89. PubMedhttp://dx.doi.org/10.1007/s007050170002

7. Ma MJ, Chen SH, Wang GL, Zhao T, Qian YH, Wu MN, et al. Novel highly pathogenic avian H5 influenza A viruses in live poultry markets, Wuxi City, China, 2013–2014. Open Forum Infect Dis. 2016;3:ofw054. PubMed http://dx.doi.org/10.1093/ofid/ofw054

Technical Appendix Table 1. Demographic characteristics of study participants, eastern China, 2013–2016*

	2013				2014				2015				2016			
Characteristic	Poultry workers	Swine workers	Controls	p value	Poultry workers	Swine workers	Controls	p value	Poultry workers	Swine workers	Controls	p value	Poultry workers	Swine workers	Controls	p value
Enrollees, no.																
Total	511	569	915		533	589	881		535	501	855		491	367	785	
Re-enrollees	NA	NA	NA		294	294	549		369	340	579		443	313	763	
New enrollees	NA	NA	NA		239	295	332		166	161	276		48	54	22	
Age, y, mean ± SD	46.8 ± 11.3	45.4 ± 10.4	41.6 ± 11.0	<0.001	48.6 ± 11.1	46.5 ± 11.3	42.6 ± 10.8	<0.001	47.9 ± 11.4	47.0 ± 11.6	42.9 ± 10.6	<0.001	49.4 ± 10.9	48.0 ± 10.2	44.1 ± 10.5	<0.001
Age group, y																
18–30	47 (9.2)	56 (9.8)	158 (17.3)	<0.001	31 (5.8)	58 (9.8)	129 (14.6)	<0.001	48 (9.0)	48 (9.6)	116 (13.5)	<0.001	32 (6.5)	21 (5.6)	94 (12.0)	<0.001
31–40	80 (15.7)	97 (17.1)	229 (25.0)		71 (13.3)	91 (15.5)	208 (23.6)		70 (13.1)	65 (13.0)	200 (23.4)		61 (12.4)	42 (11.4)	176 (22.4)	
41–50	206 (40.3)	255 (44.8)	354 (38.7)		217 (40.7)	246 (41.8)	353 (40.1)		204 (38.1)	214 (42.6)	345 (40.3)		181 (36.9)	172 (47.1)	309 (39.4)	
51–60	120 (23.4)	119 (20.9)	140 (15.3)		132 (24.8)	128 (21.7)	158 (17.9)		137 (25.6)	111 (22.2)	155 (18.2)		131 (26.7)	97 (26.4)	154 (19.6)	
>60	58 (11.4)	42 (7.4)	34 (3.7)		82 (15.4)	66 (11.2)	33 (3.8)		76 (14.2)	63 (12.6)	39 (4.6)		86 (17.5)	35 (9.5)	52 (6.6)	
Sex, no. (%)																
M	251 (49.1)	348 (61.2)	413 (45.1)	<0.001	256 (48.0)	379 (64.4)	407 (46.2)	<0.001	253 (47.3)	318 (63.5)	447 (52.3)	<0.001	235 (47.9)	237 (64.6)	399 (50.8)	<0.001
F	260 (50.9)	221 (38.8)	502 (54.9)		277 (52.0)	210 (35.6)	474 (53.8)		282 (52.7)	183 (36.5)	408 (47.7)		256 (52.1)	130 (35.4)	386 (49.2)	
Education, no. (%)																
≤Primary school	185 (36.2)	228 (40.1)	244 (26.7)	<0.001	209 (39.2)	215 (36.5)	252 (28.6)	<0.001	196 (36.6)	176 (35.1)	241 (28.2)	<0.001	176 (35.9)	140 (38.1)	219 (27.9)	<0.001
Middle school	305 (59.7)	327 (57.5)	579 (63.3)		312 (58.5)	359 (61.0)	541 (61.4)		331 (61.9)	311 (62.1)	543 (63.5)		303 (61.7)	222 (60.5)	494 (62.9)	
≥College	21 (4.1)	14 (2.5)	92 (10.0)		12 (2.3)	15 (2.5)	88 (10.0)		8 (1.5)	14 (2.8)	71 (8.3)		12 (2.4)	5 (1.4)	72 (9.2)	
Monthly cost of food, no. (%)†																
<500	29 (5.8)	50 (8.8)	41 (4.5)	<0.001	44 (8.3)	68 (11.7)	41 (4.7)	<0.001	28 (5.3)	33 (6.7)	33 (3.9)	0.002	22 (4.5)	14 (3.9)	26 (3.3)	0.003
500–1,000	152 (30.6)	210 (37.0)	161 (17.8)		169 (32.1)	170 (29.3)	217 (24.7)		149 (28.1)	159 (32.4)	214 (25.1)		133 (27.3)	85 (23.5)	191 (24.4)	
1,000–1,500	175 (35.2)	177 (31.2)	372 (41.2)		194 (36.8)	208 (35.9)	304 (34.7)		197 (37.1)	177 (36.0)	310 (36.4)		206 (42.2)	120 (33.2)	288 (36.7)	
<1,500	141 (28.4)	130 (22.9)	330 (36.5)		120 (22.8)	134 (23.1)	315 (35.9)		157 (29.6)	122 (24.8)	295 (34.6)		127 (26.0)	142 (39.3)	279 (35.6)	
Influenza vaccination, no. (%)†																
Yes	10 (2.0)	16 (2.9)	14 (1.6)	0.23	2 (0.4)	3 (0.5)	3 (0.3)	0.90	9 (1.7)	2 (0.4)	1 (0.1)	0.002	5 (1.0)	2 (0.5)	1 (0.1)	0.06
No	492 (98.0)	541 (97.1)	878 (98.4)		525 (99.6)	584 (99.5)	875 (99.7)		512 (98.3)	497 (99.6)	847 (99.9)		477 (99.0)	365 (99.5)	782 (99.9)	
Tobacco use, no. (%)†																

	2013				2014				2015				2016			
Characteristic	Poultry workers	Swine workers	Controls	p value	Poultry workers	Swine workers	Controls	p value	Poultry workers	Swine workers	Controls	p value	Poultry workers	Swine workers	Controls	p value
Yes	143 (28.0)	190 (33.4)	212 (23.2)	<0.001	134 (25.3)	210 (35.8)	193 (21.9)	<0.001	118 (22.3)	168 (34.0)	208 (25.0)	<0.001	107 (22.2)	119 (32.5)	182 (23.4)	<0.001
No	368 (72.0)	379 (66.6)	703 (76.8)		396 (74.7)	377 (64.2)	688 (78.1)		412 (77.7)	326 (66.0)	624 (75.0)		374 (77.8)	247 (67.5)	597 (76.6)	
ILI in past 12 mo, no. (%)†																
Yes	180 (35.8)	168 (29.7)	216 (23.7)	<0.001	173 (32.7)	192 (32.8)	283 (32.2)	0.96	155 (29.0)	177 (35.5)	275 (32.3)	0.08	126 (25.7)	111 (30.2)	260 (33.1)	0.02
No	323 (64.2)	397 (70.3)	695 (76.3)		356 (67.3)	394 (67.2)	597 (67.8)		379 (71.0)	321 (64.5)	577 (67.7)		364 (74.3)	256 (69.8)	525 (66.9)	
ILI of family members, no. (%)†																
Yes	144 (28.6)	105 (18.8)	170 (18.7)	<0.001	144 (27.8)	165 (28.1)	225 (25.8)	0.56	115 (21.5)	127 (25.4)	207 (24.5)	0.30	97 (19.8)	75 (20.5)	203 (26.0)	0.02
No	359 (71.4)	454 (81.2)	738 (81.3)		374 (72.2)	422 (71.9)	647 (74.2)		419 (78.5)	373 (74.6)	639 (75.5)		394 (80.2)	291 (79.5)	578 (74.0)	

*ILI, influenza-like illness; NA, not available.
†Variable has missing data; The unit of cost is Chinses RMB.

Technical Appendix Table 2. Characteristic of participants with seropositive for influenza A(H7N9), A(H9N2), A(H5N1), and A(H5N6) viruses, eastern China, 2013–2016*

Virus, participant no.	Age, y/sex	Occupation	Chronic medical condition	ILI in past 12 mo	MN titer			
					2013	2014	2015	2016
H7N9								
1	28/F	Chicken slaughtering	No	Yes	40	320	5	5
2	41/F	Chicken slaughtering	No	No	5	5	320	NA
3	63/F	Retired (Control)	Hypertension	No	20	80	NA	NA
31	50/F	Chicken slaughtering	No	No	NA	320	NA	NA
32	26/F	Chicken slaughtering	No	Yes	NA	80	NA	NA
33	51/F	Chicken slaughtering	No	No	320	5	5	5
34	45/M	Chicken seller	Chronic bronchitis	No	160	5	5	5
35	49/F	Chicken/duck seller	No	No	NA	NA	160	5
36	40/M	Chicken/pigeon seller	No	Yes	NA	NA	160	5
37	42/M	Chicken slaughtering	No	No	160	NA	NA	NA
38	61/M	Pig slaughtering	No	No	NA	160	NA	NA
39	59/M	Pork seller	No	No	NA	NA	NA	160
40	49/F	Officer (Control)	No	Yes	80	NA	NA	NA
41	46/F	Officer (Control)	No	No	80	NA	NA	NA
42	57/F	Officer (Control)	No	No	NA	NA	NA	80
H9N2								
4	48/F	Chicken backyard grower	No	No	5	5	80	40
5	28/M	Chicken raising	No	No	NA	5	80	80
6	51/F	Chicken raising	No	No	5	5	5	80
7	47/F	Chicken seller	No	No	5	20	80	40
8	47/M	Chicken seller	No	No	5	5	160	NA
9	46/M	Chicken seller	No	No	5	5	160	NA
10	51/M	Chicken seller	Chronic bronchitis	No	5	40	160	NA
11	49/M	Chicken/duck seller	Diabetes	No	NA	NA	20	80
12	59/F	Chicken/duck seller	No	Yes	5	5	80	320
13	39/F	Chicken/duck seller	No	No	5	NA	20	80
14	27/F	Chicken/goose seller	No	No	5	320	40	40
15	57/F	Chicken/pigeon slaughtering	No	No	5	40	80	5
16	52/F	Duck/goose seller	No	No	5	80	5	5
17	32/M	Pig slaughtering	No	No	5	5	5	80
43	36/F	Chicken slaughtering	No	No	160	5	5	5
44	42/M	Chicken seller	No	No	NA	NA	80	NA
45	29/M	Chicken seller	No	No	NA	NA	80	80
46	39/M	Chicken seller	No	No	NA	NA	80	NA
47	46/F	Chicken/duck seller	No	No	NA	NA	NA	160
18	52/M	Pig slaughtering	No	No	5	80	NA	80
19	26/M	Pork seller	No	No	5	5	5	160
20	40/M	Grocer (Control)	Hypertension	No	5	160	5	5
21	48/M	Grocer (Control)	No	No	5	5	80	5
22	38/M	Grocer (Control)	Diabetes	Yes	5	5	160	5
23	61/M	Retired (Control)	No	No	NA	5	5	80
48	48/M	Grocer (Control)	No	No	NA	NA	80	5
49	49/M	Officer (Control)	No	Yes	NA	NA	160	5
50	39/M	Grocer (Control)	No	No	160	NA	20	NA
51	52/F	Officer (Control)	No	No	NA	NA	NA	80
52	61/M	Retired (Control)	No	No	80	40	NA	NA
H5N1								
24	39/F	Chicken/duck/goose seller	No	No	5	5	20	80
25	45/F	Chicken/duck/pigeon raising	No	No	20	10	40	80
26	48/M	Pigeon seller	No	No	10	10	10	80
27	60/F	Chicken/goose seller	No	No	10	5	40	80
28	55/F	Duck/goose seller	No	No	5	5	40	160
29	46/F	Chicken slaughtering	No	No	40	20	20	80
30	53/F	Chicken slaughtering	No	No	20	5	20	80
53	28/F	Chicken/goose seller	No	No	80	5	NA	NA
54	55/F	Chicken processing	No	No	5	5	5	80
55	46/M	Chicken raising	No	No	NA	NA	NA	80
56	67/M	Chicken seller	No	No	NA	40	40	80
57	47/F	Chicken/duck raising	No	No	NA	NA	NA	160
58	66/F	Chicken/duck seller	No	No	NA	NA	NA	80
59	58/F	Chicken/duck/pigeon seller	Chronic bronchitis	No	NA	NA	40	80
60	46/F	Chicken/duck/pigeon seller	No	No	NA	NA	NA	80
61	51/M	Chicken/goose raising	No	No	40	NA	NA	80
62	57/F	Pigeon raising	No	No	NA	NA	40	80
63	57/F	Pigeon raising	No	No	5	40	5	80

*F, female; M, male; ILI, influenza-like Illness; MN, microneutralization; NA, the participant was not available in this year.

Technical Appendix Table 3. Characterization of selected molecular markers associated with infectivity, pathogenicity and antiviral susceptibility of viruses identified in the present study, eastern China, 2013–2016

	HA						NA		M2	PB2	
Virus name	D158N	T160A	E190D	Q226L	G228S	Cleavage	R292K	Deletion	S31N	E627K	D701N
A/chicken/Wuxi/5852/2015(H7N9) *	N	A	E	L	G	EIPKGR/G	R	69–73	N	E	D
A/chicken/Wuxi/7144/2015(H7N9) *	N	A	E	L	G	EIPKGR/G	R	69–73	N	E	D
A/chicken/Wuxi/8048/2016(H7N9) *	N	A	E	L	G	EIPKGR/G	R	69–73	N	E	D
A/chicken/Wuxi/WX5/2014(H7N9) *	N	A	E	L	G	EIPKGR/G	R	69–73	N	E	D
A/environment/Wuxi/Hu*n/2014(H7N9) *	N	A	E	Q	G	EIPKGR/G	R	69–73	N	E	D
A/environment/Wuxi/WA021/2013(H9N2) *	N	N	T	L	G	PSRSSR/G	K	63–65	N	E	D
A/environment/Wuxi/1062/2013(H9N2) *	N	D	T	L	G	PSPFSR/G	K	63–65	N	E	D
A/environment/Wuxi/2505/2014(H9N2) *	N	D	T	L	G	PSRSSR/G	K	63–65	N	E	D
A/duck/Wuxi/5083/2015(H9N2) *	N	D	T	L	G	PSRSSR/G	K	63–65	N	E	D
A/environment/Wuxi/5220/2015(H9N2) *	N	D	T	L	G	PSRSSR/G	K	63–65	N	E	D
A/goose/Wuxi/5842/2015(H9N2) *	N	D	T	L	G	PSRSSR/G	K	63–65	N	E	D
A/chicken/Wuxi/6082/2015(H9N2) *	N	D	T	L	G	PSRSSR/G	K	63–65	N	E	D
A/chicken/Wuxi/6085/2015(H9N2) *	N	D	T	L	G	PSRSSR/G	K	63–65	N	E	D
A/chicken/Wuxi/6088/2015(H9N2) *	N	D	T	L	G	PSRSSR/G	K	63–65	N	E	D
A/chicken/Wuxi/6224/2015(H9N2) *	N	D	T	L	G	PSRSSR/G	K	63–65	N	E	D
A/chicken/Wuxi/6414/2015(H9N2) *	N	D	T	L	G	PSRSSR/G	K	63–65	N	E	D
A/chicken/Wuxi/6440/2015(H9N2) *	N	D	T	L	G	PSRSSR/G	K	63–65	N	E	D
A/chicken/Wuxi/6442/2015(H9N2) *	N	D	T	L	G	PSRSSR/G	K	63–65	N	E	D
A/chicken/Wuxi/6468/2015(H9N2) *	N	D	T	L	G	PSRSSR/G	K	63–65	N	E	D
A/chicken/Wuxi/6650/2015(H9N2) *	N	D	T	L	G	PSRSSR/G	K	63–65	N	E	D
A/chicken/Wuxi/6657/2015(H9N2) *	N	D	T	L	G	PSRSSR/G	K	63–65	N	E	D
A/duck/Wuxi/6659/2015(H9N2) *	N	D	T	L	G	PSRSSR/G	K	63–65	N	E	D
A/duck/Wuxi/6663/2015(H9N2) *	N	D	T	L	G	PSRSSR/G	K	63–65	N	E	D
A/chicken/Wuxi/6688/2015(H9N2) *	N	D	T	L	G	PSPFSR/G	K	63–65	N	E	D
A/chicken/Wuxi/7022/2015(H9N2) *	N	D	T	L	G	PSRSSR/G	K	63–65	N	E	D
A/chicken/Wuxi/7109/2015(H9N2) *	N	D	T	L	G	PSRSSR/G	K	63–65	N	E	D
A/chicken/Wuxi/6808/2015(H9N2) *	N	D	T	L	G	PSRSSR/G	K	63–65	N	E	D
A/chicken/Wuxi7723/2016(H9N2) *	N	A	T	L	G	PSRSSR/G	K	63–65	N	E	D
A/chicken/Wuxi/8500/2016(H9N2) *	N	E	T	L	G	PSRSSR/G	K	63–65	N	E	D
A/chicken/Wuxi/2723/2014(H9N2)	N	D	T	L	G	PSRSSR/G	K	63–65	N	NSD	NSD
A/chicken/Wuxi/3083/2014(H9N2)	N	D	T	L	G	PSPFSR/G	K	63–65	N	NSD	NSD
A/chicken/Wuxi/3085/2014(H9N2)	N	D	T	L	G	PSPFSR/G	K	63–65	N	NSD	NSD
A/chicken/Wuxi/5854/2015(H9N2)	N	D	T	L	G	PSRSSR/G	K	63–65	N	NSD	NSD
A/pigeon/Wuxi/5997/2015(H9N2)	N	D	T	L	G	PSRSSR/G	K	63–65	N	NSD	NSD
A/pigeon/Wuxi/5998/2015(H9N2)	N	D	T	L	G	PSPFSR/G	K	63–65	N	NSD	NSD
A/chicken/Wuxi/5999/2015(H9N2)	N	D	T	L	G	PSPFSR/G	K	63–65	N	NSD	NSD
A/chicken/Wuxi/6080/2015(H9N2)	N	D	T	L	G	PSRSSR/G	K	63–65	N	NSD	NSD
A/chicken/Wuxi/6084/2015(H9N2)	N	D	T	L	G	PSRSSR/G	K	63–65	N	NSD	NSD
A/chicken/Wuxi/6225/2015(H9N2)	N	D	T	L	G	PSRSSR/G	K	63–65	N	NSD	NSD
A/chicken/Wuxi/6424/2015(H9N2)	N	D	T	L	G	PSRSSR/G	K	63–65	N	NSD	NSD
A/chicken/Wuxi/6435/2015(H9N2)	N	D	T	L	G	PSRSSR/G	K	63–65	N	E	D
A/chicken/Wuxi/6441/2015(H9N2)	N	D	T	L	G	PSRSSR/G	K	63–65	N	NSD	NSD
A/chicken/Wuxi/6469/2015(H9N2)	N	D	T	L	G	PSRSSR/G	K	63–65	N	NSD	NSD
A/chicken/Wuxi/6638/2015(H9N2)	N	D	T	L	G	PSRSSR/G	K	63–65	N	NSD	NSD

Virus name	HA						NA		M2	PB2	
	D158N	T160A	E190D	Q226L	G228S	Cleavage	R292K	Deletion	S31N	E627K	D701N
A/chicken/Wuxi/6643/2015(H9N2)	N	D	T	L	G	PSRSSR/G	K	63–65	N	NSD	NSD
A/duck/Wuxi/6644/2015(H9N2)	N	D	T	L	G	PSRSSR/G	K	63–65	N	NSD	NSD
A/chicken/Wuxi/6649/2015(H9N2)	N	D	T	L	G	PSRSSR/G	K	63–65	N	NSD	NSD
A/chicken/Wuxi/7107/2015(H9N2)	N	D	T	L	G	PSRSSR/G	K	63–65	N	E	D
A/chicken/Wuxi/7124/2015(H9N2)	N	D	T	L	G	PSRSSR/G	K	63–65	N	NSD	NSD
A/chicken/Wuxi/7130/2015(H9N2)	N	D	T	L	G	PSRSSR/G	K	63–65	N	NSD	NSD
A/chicken/Wuxi/7346/2015(H9N2)	D	D	T	L	G	PSPSSR/G	K	63–65	N	NSD	NSD
A/chicken/Wuxi/7641/2016(H9N2)	N	E	T	L	G	PSRSSR/G	K	63–65	N	NSD	NSD
A/chicken/Wuxi/7157/2015(H5N6) *	N	A	E	Q	G	ERRRKR/G	R	58–68	S	E	D
A/duck/Wuxi/7249/2015(H5N6) *	N	A	E	Q	G	ERRRKR/G	R	58–68	S	E	D
A/chicken/Wuxi/7765/2016(H5N6) *	N	A	E	Q	G	ERRRKR/G	R	58–68	S	E	D
A/environment/Wuxi/1275/2014(H5N1)	D	A	E	Q	G	ERRRKR/G	R	49–68	S	NSD	NSD
A/environment/Wuxi/4689/2015(H5N1)	N	T	E	Q	G	ERRRKR/G	R	49–68	N	NSD	NSD
A/environment/Wuxi/5068/2015(H5N1)	N	T	E	Q	G	ERRRKR/G	R	49–68	S	NSD	NSD
A/environment/Wuxi/5081/2015(H5N1)	D	A	E	Q	G	ERRRKR/G	R	49–68	N	NSD	NSD
A/chicken/Wuxi/6074/2015(H5N2)	N	A	E	Q	G	ERRRKR/G	R	No	N	NSD	NSD
A/duck/Wuxi/6462/2015(H5N2)	N	A	E	Q	G	ERRRKR/G	R	No	N	NSD	NSD
A/duck/Wuxi/6466/2015(H5N2)	N	A	E	Q	G	ERRRKR/G	R	No	N	NSD	NSD
A/chicken/Wuxi/2722/2014(H5N2)	N	T	E	Q	G	ERRRKR/G	R	No	N	NSD	NSD
A/environment/Wuxi/1772/2014(H5N2)	N	T	E	Q	G	ERRRKR/G	R	No	N	NSD	NSD
A/chicken/Wuxi/JYJN132/2014(H11N2) *	S	T	E	Q	G	PAIASR/G	R	No	S	E	D
A/duck/Wuxi/JYJN126/2014(H11N2) *	S	T	E	Q	G	PAIASR/G	R	No	S	E	D
A/duck/Wuxi/JYJN203/2014(H11N2) *	S	T	E	Q	G	PAIASR/G	R	No	S	E	D
A/chicken/Wuxi/4859/2015(H3N8) *	G	A	E	Q	G	PEKQTR/G	R	No	S	E	D
A/duck/Wuxi/7275/2016(H3N8) *	G	A	E	Q	G	PEKQTR/G	R	No	S	E	D
A/goose/Wuxi/7276/2015(H3N8) *	G	A	E	Q	G	PEKQTR/G	R	No	S	E	D
A/chicken/Wuxi/5682/2015(H1N1)	G	S	E	Q	G	PSIQSR/G	R	63–65	N	E	NSD

*Viral isolation was successful. HA, hemagglutinin; NA, neuraminidase; M, matrix; PB2, polymerase basic 2; NSD, no sequence data.

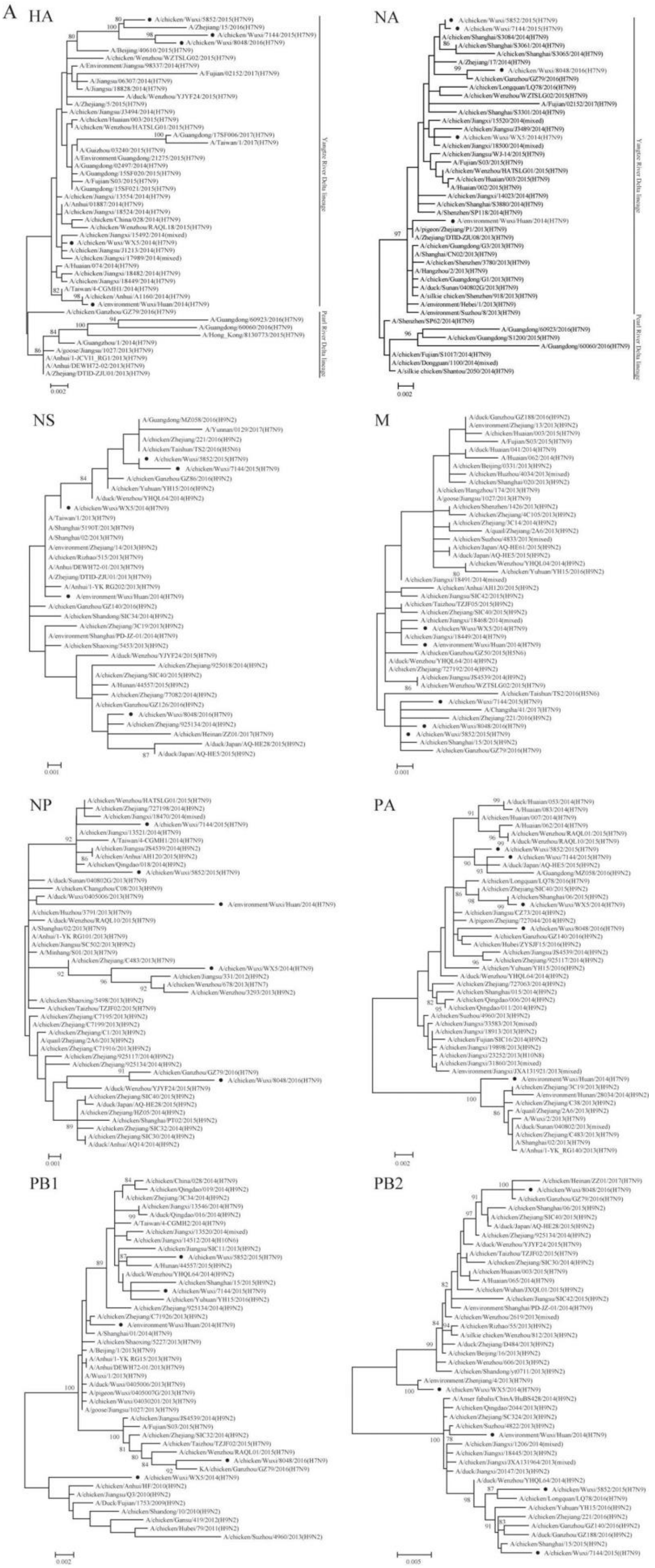
A
HA
NA
NS
M
NP
PA
PB1
PB2

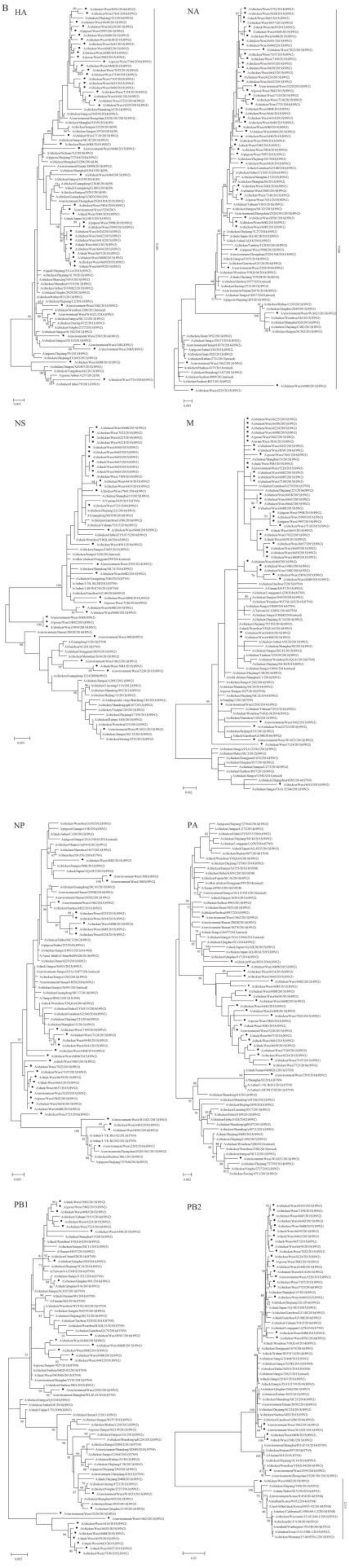
B
HA
NA
NS
M
NP
PA
PB1
PB2

HA NA BPT

C

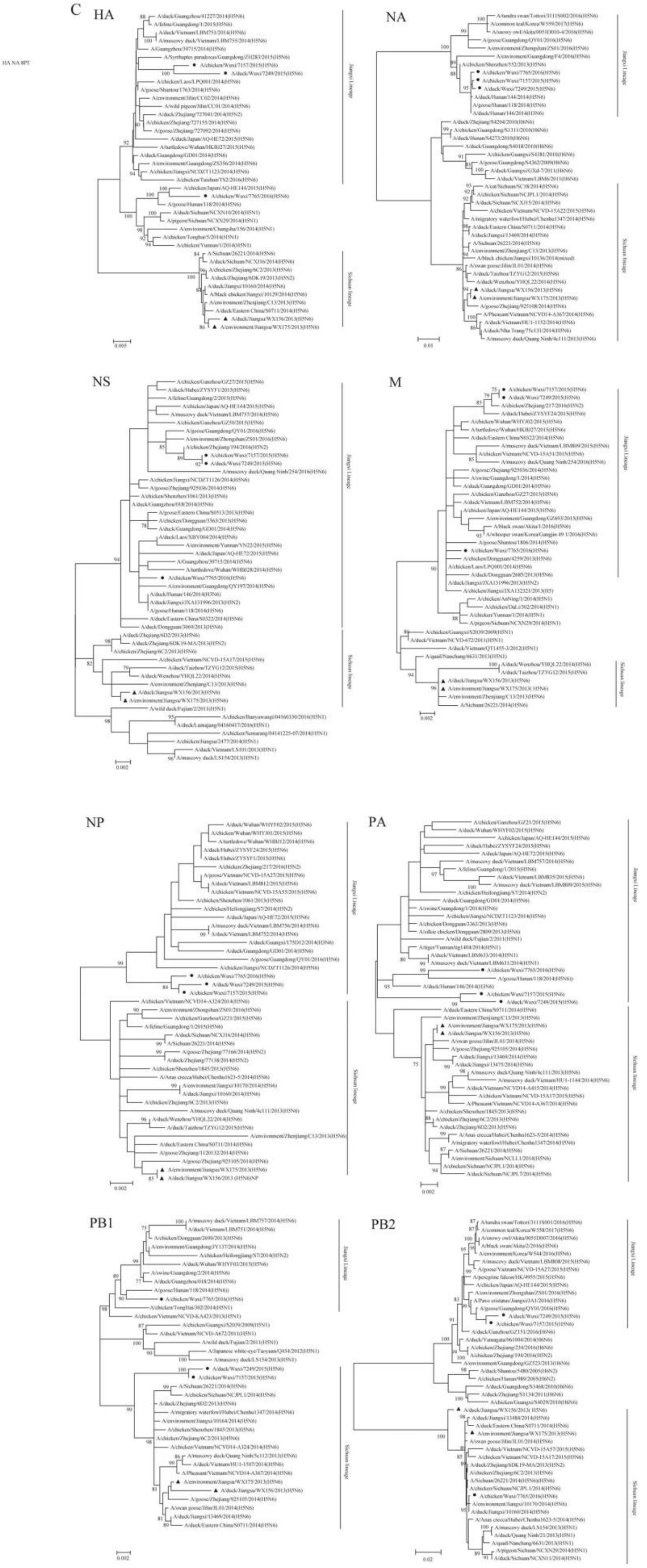

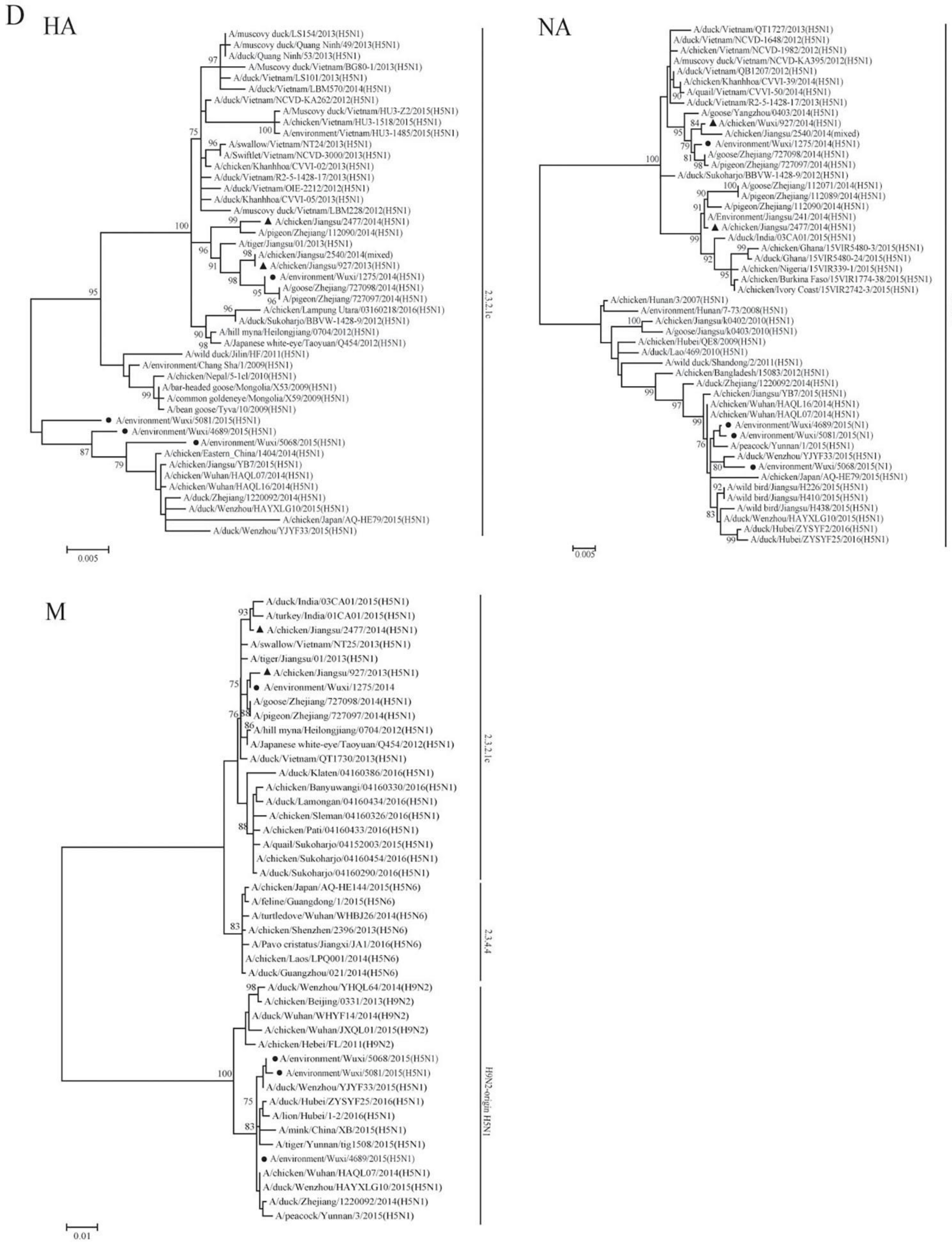

D
HA
A/muscovy duck/LS154/2013(H5N1)
A/muscovy duck/Quang Ninh/49/2013(H5N1)
A/duck/Quang Ninh/53/2013(H5N1)
A/Muscovy duck/Vietnam/BG80-1/2013(H5N1)
A/duck/Vietnam/LS101/2013(H5N1)
A/duck/Vietnam/LBM570/2014(H5N1)
A/duck/Vietnam/NCVD-KA262/2012(H5N1)
A/Muscovy duck/Vietnam/HU3-Z2/2015(H5N1)
A/chicken/Vietnam/HU3-1518/2015(H5N1)
A/environment/Vietnam/HU3-1485/2015(H5N1)
A/swallow/Vietnam/NT24/2013(H5N1)
A/Swiftlet/Vietnam/NCVD-3000/2013(H5N1)
A/chicken/Khanhhoa/CVVI-02/2013(H5N1)
A/duck/Vietnam/R2-5-1428-17/2013(H5N1)
A/duck/Vietnam/OIE-2212/2012(H5N1)
A/duck/Khanhhoa/CVVI-05/2013(H5N1)
A/muscovy duck/Vietnam/LBM228/2012(H5N1)
A/chicken/Jiangsu/2477/2014(H5N1)
A/pigeon/Zhejiang/112090/2014(H5N1)
A/tiger/Jiangsu/01/2013(H5N1)
A/chicken/Jiangsu/2540/2014(mixed)
A/chicken/Jiangsu/927/2013(H5N1)
A/environment/Wuxi/1275/2014(H5N1)
A/goose/Zhejiang/727098/2014(H5N1)
A/pigeon/Zhejiang/727097/2014(H5N1)
A/chicken/Lampung Utara/03160218/2016(H5N1)
A/duck/Sukoharjo/BBVW-1428-9/2012(H5N1)
A/hill myna/Heilongjiang/0704/2012(H5N1)
A/Japanese white-eye/Taoyuan/Q454/2012(H5N1)
A/wild duck/Jilin/HF/2011(H5N1)
A/environment/Chang Sha/1/2009(H5N1)
A/chicken/Nepal/5-1el/2010(H5N1)
A/bar-headed goose/Mongolia/X53/2009(H5N1)
A/common goldeneye/Mongolia/X59/2009(H5N1)
A/bean goose/Tyva/10/2009(H5N1)
A/environment/Wuxi/5081/2015(H5N1)
A/environment/Wuxi/4689/2015(H5N1)
A/environment/Wuxi/5068/2015(H5N1)
A/chicken/Eastern_China/1404/2014(H5N1)
A/chicken/Jiangsu/YB7/2015(H5N1)
A/chicken/Wuhan/HAQL07/2014(H5N1)
A/chicken/Wuhan/HAQL16/2014(H5N1)
A/duck/Zhejiang/1220092/2014(H5N1)
A/duck/Wenzhou/HAYXLG10/2015(H5N1)
A/chicken/Japan/AQ-HE79/2015(H5N1)
A/duck/Wenzhou/YJYF33/2015(H5N1)
2.3.2.1c
0.005
NA
A/duck/Vietnam/QT1727/2013(H5N1)
A/duck/Vietnam/NCVD-1648/2012(H5N1)
A/chicken/Vietnam/NCVD-1982/2012(H5N1)
A/muscovy duck/Vietnam/NCVD-KA395/2012(H5N1)
A/duck/Vietnam/QB1207/2012(H5N1)
A/chicken/Khanhhoa/CVVI-39/2014(H5N1)
A/quail/Vietnam/CVVI-50/2014(H5N1)
A/duck/Vietnam/R2-5-1428-17/2013(H5N1)
A/goose/Yangzhou/0403/2014(H5N1)
A/chicken/Wuxi/927/2014(H5N1)
A/chicken/Jiangsu/2540/2014(mixed)
A/environment/Wuxi/1275/2014(H5N1)
A/goose/Zhejiang/727098/2014(H5N1)
A/pigeon/Zhejiang/727097/2014(H5N1)
A/duck/Sukoharjo/BBVW-1428-9/2012(H5N1)
A/goose/Zhejiang/112071/2014(H5N1)
A/pigeon/Zhejiang/112089/2014(H5N1)
A/pigeon/Zhejiang/112090/2014(H5N1)
A/Environment/Jiangsu/241/2014(H5N1)
A/chicken/Jiangsu/2477/2014(H5N1)
A/duck/India/03CA01/2015(H5N1)
A/chicken/Ghana/15VIR5480-3/2015(H5N1)
A/duck/Ghana/15VIR5480-24/2015(H5N1)
A/chicken/Nigeria/15VIR339-1/2015(H5N1)
A/chicken/Burkina Faso/15VIR1774-38/2015(H5N1)
A/chicken/Ivory Coast/15VIR2742-3/2015(H5N1)
A/chicken/Hunan/3/2007(H5N1)
A/environment/Hunan/7-73/2008(H5N1)
A/chicken/Jiangsu/k0402/2010(H5N1)
A/goose/Jiangsu/k0403/2010(H5N1)
A/chicken/Hubei/QE8/2009(H5N1)
A/duck/Lao/469/2010(H5N1)
A/wild duck/Shandong/2/2011(H5N1)
A/chicken/Bangladesh/15083/2012(H5N1)
A/duck/Zhejiang/1220092/2014(H5N1)
A/chicken/Jiangsu/YB7/2015(H5N1)
A/chicken/Wuhan/HAQL16/2014(H5N1)
A/chicken/Wuhan/HAQL07/2014(H5N1)
A/environment/Wuxi/4689/2015(N1)
A/environment/Wuxi/5081/2015(N1)
A/peacock/Yunnan/1/2015(H5N1)
A/duck/Wenzhou/YJYF33/2015(H5N1)
A/environment/Wuxi/5068/2015(N1)
A/chicken/Japan/AQ-HE79/2015(H5N1)
A/wild bird/Jiangsu/H226/2015(H5N1)
A/wild bird/Jiangsu/H410/2015(H5N1)
A/wild bird/Jiangsu/H438/2015(H5N1)
A/duck/Wenzhou/HAYXLG10/2015(H5N1)
A/duck/Hubei/ZYSYF2/2016(H5N1)
A/duck/Hubei/ZYSYF25/2016(H5N1)
2.3.2.1c
0.005
M
A/duck/India/03CA01/2015(H5N1)
A/turkey/India/01CA01/2015(H5N1)
A/chicken/Jiangsu/2477/2014(H5N1)
A/swallow/Vietnam/NT25/2013(H5N1)
A/tiger/Jiangsu/01/2013(H5N1)
A/chicken/Jiangsu/927/2013(H5N1)
A/environment/Wuxi/1275/2014
A/goose/Zhejiang/727098/2014(H5N1)
A/pigeon/Zhejiang/727097/2014(H5N1)
A/hill myna/Heilongjiang/0704/2012(H5N1)
A/Japanese white-eye/Taoyuan/Q454/2012(H5N1)
A/duck/Vietnam/QT1730/2013(H5N1)
A/duck/Klaten/04160386/2016(H5N1)
A/chicken/Banyuwangi/04160330/2016(H5N1)
A/duck/Lamongan/04160434/2016(H5N1)
A/chicken/Sleman/04160326/2016(H5N1)
A/chicken/Pati/04160433/2016(H5N1)
A/quail/Sukoharjo/04152003/2015(H5N1)
A/chicken/Sukoharjo/04160454/2016(H5N1)
A/duck/Sukoharjo/04160290/2016(H5N1)
2.3.2.1c
A/chicken/Japan/AQ-HE144/2015(H5N6)
A/feline/Guangdong/1/2015(H5N6)
A/turtledove/Wuhan/WHBJ26/2014(H5N6)
A/chicken/Shenzhen/2396/2013(H5N6)
A/Pavo cristatus/Jiangxi/JA1/2016(H5N6)
A/chicken/Laos/LPQ001/2014(H5N6)
A/duck/Guangzhou/021/2014(H5N6)
2.3.4.4
A/duck/Wenzhou/YHQL64/2014(H9N2)
A/chicken/Beijing/0331/2013(H9N2)
A/duck/Wuhan/WHYF14/2014(H9N2)
A/chicken/Wuhan/JXQL01/2015(H9N2)
A/chicken/Hebei/FL/2011(H9N2)
A/environment/Wuxi/5068/2015(H5N1)
A/environment/Wuxi/5081/2015(H5N1)
A/duck/Wenzhou/YJYF33/2015(H5N1)
A/duck/Hubei/ZYSYF25/2016(H5N1)
A/lion/Hubei/1-2/2016(H5N1)
A/mink/China/XB/2015(H5N1)
A/tiger/Yunnan/tig1508/2015(H5N1)
A/environment/Wuxi/4689/2015(H5N1)
A/chicken/Wuhan/HAQL07/2014(H5N1)
A/duck/Wenzhou/HAYXLG10/2015(H5N1)
A/duck/Zhejiang/1220092/2014(H5N1)
A/peacock/Yunnan/3/2015(H5N1)
H9N2-origin H5N1
0.01

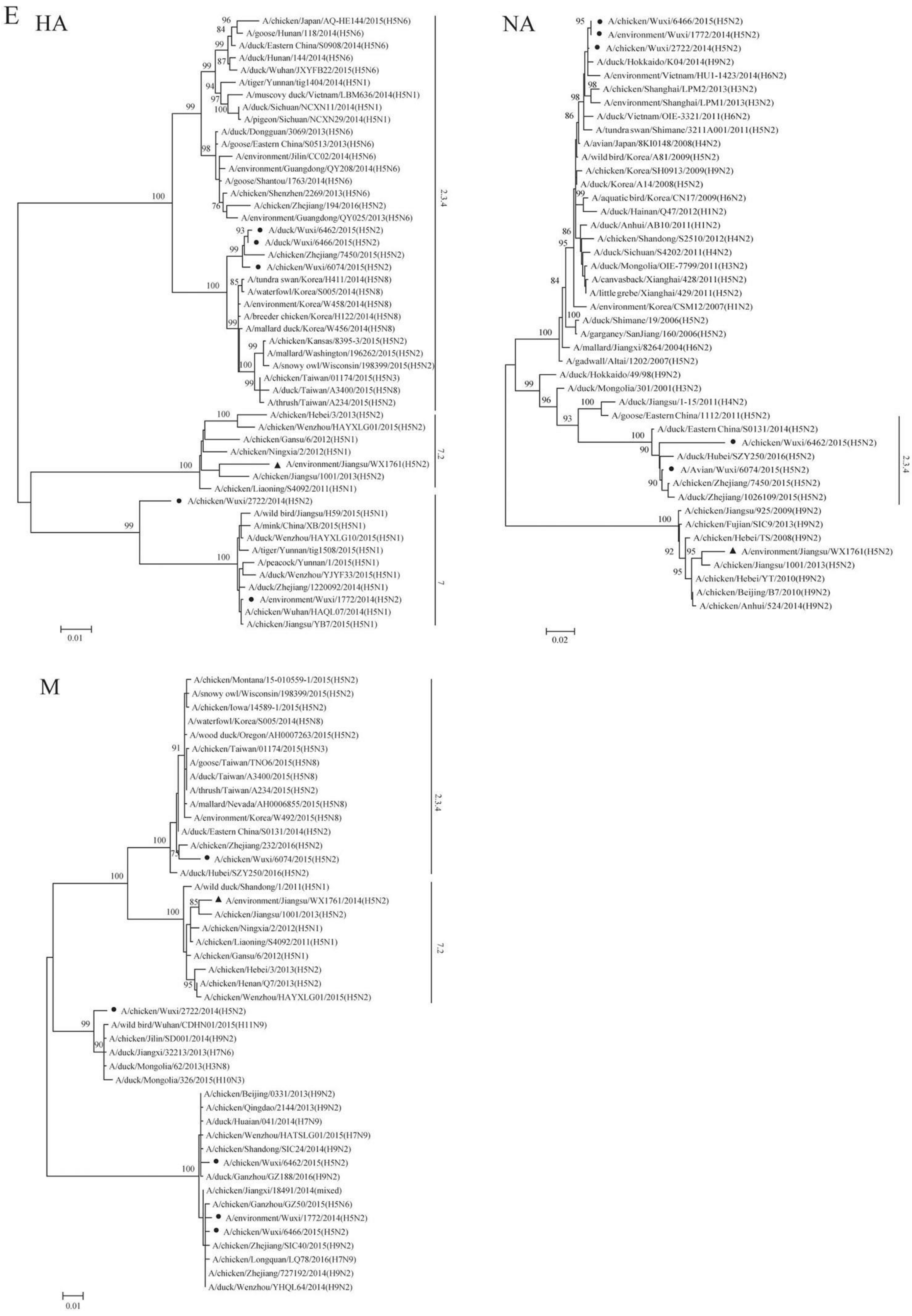

E
HA
A/chicken/Japan/AQ-HE144/2015(H5N6)
A/goose/Hunan/118/2014(H5N6)
A/duck/Eastern China/S0908/2014(H5N6)
A/duck/Hunan/144/2014(H5N6)
A/duck/Wuhan/JXYFB22/2015(H5N6)
A/tiger/Yunnan/tig1404/2014(H5N1)
A/muscovy duck/Vietnam/LBM636/2014(H5N1)
A/duck/Sichuan/NCXN11/2014(H5N1)
A/pigeon/Sichuan/NCXN29/2014(H5N1)
A/duck/Dongguan/3069/2013(H5N6)
A/goose/Eastern China/S0513/2013(H5N6)
A/environment/Jilin/CC02/2014(H5N6)
A/environment/Guangdong/QY208/2014(H5N6)
A/goose/Shantou/1763/2014(H5N6)
A/chicken/Shenzhen/2269/2013(H5N6)
A/chicken/Zhejiang/194/2016(H5N2)
A/environment/Guangdong/QY025/2013(H5N6)
A/duck/Wuxi/6462/2015(H5N2)
A/duck/Wuxi/6466/2015(H5N2)
A/chicken/Zhejiang/7450/2015(H5N2)
A/chicken/Wuxi/6074/2015(H5N2)
A/tundra swan/Korea/H411/2014(H5N8)
A/waterfowl/Korea/S005/2014(H5N8)
A/environment/Korea/W458/2014(H5N8)
A/breeder chicken/Korea/H122/2014(H5N8)
A/mallard duck/Korea/W456/2014(H5N8)
A/chicken/Kansas/8395-3/2015(H5N2)
A/mallard/Washington/196262/2015(H5N2)
A/snowy owl/Wisconsin/198399/2015(H5N2)
A/chicken/Taiwan/01174/2015(H5N3)
A/duck/Taiwan/A3400/2015(H5N8)
A/thrush/Taiwan/A234/2015(H5N2)
2.3.4
A/chicken/Hebei/3/2013(H5N2)
A/chicken/Wenzhou/HAYXLG01/2015(H5N2)
A/chicken/Gansu/6/2012(H5N1)
A/chicken/Ningxia/2/2012(H5N1)
A/environment/Jiangsu/WX1761(H5N2)
A/chicken/Jiangsu/1001/2013(H5N2)
A/chicken/Liaoning/S4092/2011(H5N1)
7.2
A/chicken/Wuxi/2722/2014(H5N2)
A/wild bird/Jiangsu/H59/2015(H5N1)
A/mink/China/XB/2015(H5N1)
A/duck/Wenzhou/HAYXLG10/2015(H5N1)
A/tiger/Yunnan/tig1508/2015(H5N1)
A/peacock/Yunnan/1/2015(H5N1)
A/duck/Wenzhou/YJYF33/2015(H5N1)
A/duck/Zhejiang/1220092/2014(H5N1)
A/environment/Wuxi/1772/2014(H5N2)
A/chicken/Wuhan/HAQL07/2014(H5N1)
A/chicken/Jiangsu/YB7/2015(H5N1)
7
0.01
NA
A/chicken/Wuxi/6466/2015(H5N2)
A/environment/Wuxi/1772/2014(H5N2)
A/chicken/Wuxi/2722/2014(H5N2)
A/duck/Hokkaido/K04/2014(H9N2)
A/environment/Vietnam/HU1-1423/2014(H6N2)
A/chicken/Shanghai/LPM2/2013(H3N2)
A/environment/Shanghai/LPM1/2013(H3N2)
A/duck/Vietnam/OIE-3321/2011(H6N2)
A/tundra swan/Shimane/3211A001/2011(H5N2)
A/avian/Japan/8KI0148/2008(H4N2)
A/wild bird/Korea/A81/2009(H5N2)
A/chicken/Korea/SH0913/2009(H9N2)
A/duck/Korea/A14/2008(H5N2)
A/aquatic bird/Korea/CN17/2009(H6N2)
A/duck/Hainan/Q47/2012(H1N2)
A/duck/Anhui/AB10/2011(H1N2)
A/chicken/Shandong/S2510/2012(H4N2)
A/duck/Sichuan/S4202/2011(H4N2)
A/duck/Mongolia/OIE-7799/2011(H3N2)
A/canvasback/Xianghai/428/2011(H5N2)
A/little grebe/Xianghai/429/2011(H5N2)
A/environment/Korea/CSM12/2007(H1N2)
A/duck/Shimane/19/2006(H5N2)
A/garganey/SanJiang/160/2006(H5N2)
A/mallard/Jiangxi/8264/2004(H6N2)
A/gadwall/Altai/1202/2007(H5N2)
A/duck/Hokkaido/49/98(H9N2)
A/duck/Mongolia/301/2001(H3N2)
A/duck/Jiangsu/1-15/2011(H4N2)
A/goose/Eastern China/1112/2011(H5N2)
A/duck/Eastern China/S0131/2014(H5N2)
A/chicken/Wuxi/6462/2015(H5N2)
A/duck/Hubei/SZY250/2016(H5N2)
A/Avian/Wuxi/6074/2015(H5N2)
A/chicken/Zhejiang/7450/2015(H5N2)
A/duck/Zhejiang/1026109/2015(H5N2)
2.3.4
A/chicken/Jiangsu/925/2009(H9N2)
A/chicken/Fujian/SIC9/2013(H9N2)
A/chicken/Hebei/TS/2008(H9N2)
A/environment/Jiangsu/WX1761(H5N2)
A/chicken/Jiangsu/1001/2013(H5N2)
A/chicken/Hebei/YT/2010(H9N2)
A/chicken/Beijing/B7/2010(H9N2)
A/chicken/Anhui/524/2014(H9N2)
0.02
M
A/chicken/Montana/15-010559-1/2015(H5N2)
A/snowy owl/Wisconsin/198399/2015(H5N2)
A/chicken/Iowa/14589-1/2015(H5N2)
A/waterfowl/Korea/S005/2014(H5N8)
A/wood duck/Oregon/AH0007263/2015(H5N2)
A/chicken/Taiwan/01174/2015(H5N3)
A/goose/Taiwan/TNO6/2015(H5N8)
A/duck/Taiwan/A3400/2015(H5N8)
A/thrush/Taiwan/A234/2015(H5N2)
A/mallard/Nevada/AH0006855/2015(H5N8)
A/environment/Korea/W492/2015(H5N8)
A/duck/Eastern China/S0131/2014(H5N2)
A/chicken/Zhejiang/232/2016(H5N2)
A/chicken/Wuxi/6074/2015(H5N2)
A/duck/Hubei/SZY250/2016(H5N2)
2.3.4
A/wild duck/Shandong/1/2011(H5N1)
A/environment/Jiangsu/WX1761/2014(H5N2)
A/chicken/Jiangsu/1001/2013(H5N2)
A/chicken/Ningxia/2/2012(H5N1)
A/chicken/Liaoning/S4092/2011(H5N1)
A/chicken/Gansu/6/2012(H5N1)
A/chicken/Hebei/3/2013(H5N2)
A/chicken/Henan/Q7/2013(H5N2)
A/chicken/Wenzhou/HAYXLG01/2015(H5N2)
7.2
A/chicken/Wuxi/2722/2014(H5N2)
A/wild bird/Wuhan/CDHN01/2015(H11N9)
A/chicken/Jilin/SD001/2014(H9N2)
A/duck/Jiangxi/32213/2013(H7N6)
A/duck/Mongolia/62/2013(H3N8)
A/duck/Mongolia/326/2015(H10N3)
A/chicken/Beijing/0331/2013(H9N2)
A/chicken/Qingdao/2144/2013(H9N2)
A/duck/Huaian/041/2014(H7N9)
A/chicken/Wenzhou/HATSLG01/2015(H7N9)
A/chicken/Shandong/SIC24/2014(H9N2)
A/chicken/Wuxi/6462/2015(H5N2)
A/duck/Ganzhou/GZ188/2016(H9N2)
A/chicken/Jiangxi/18491/2014(mixed)
A/chicken/Ganzhou/GZ50/2015(H5N6)
A/environment/Wuxi/1772/2014(H5N2)
A/chicken/Wuxi/6466/2015(H5N2)
A/chicken/Zhejiang/SIC40/2015(H9N2)
A/chicken/Longquan/LQ78/2016(H7N9)
A/chicken/Zhejiang/727192/2014(H9N2)
A/duck/Wenzhou/YHQL64/2014(H9N2)
0.01

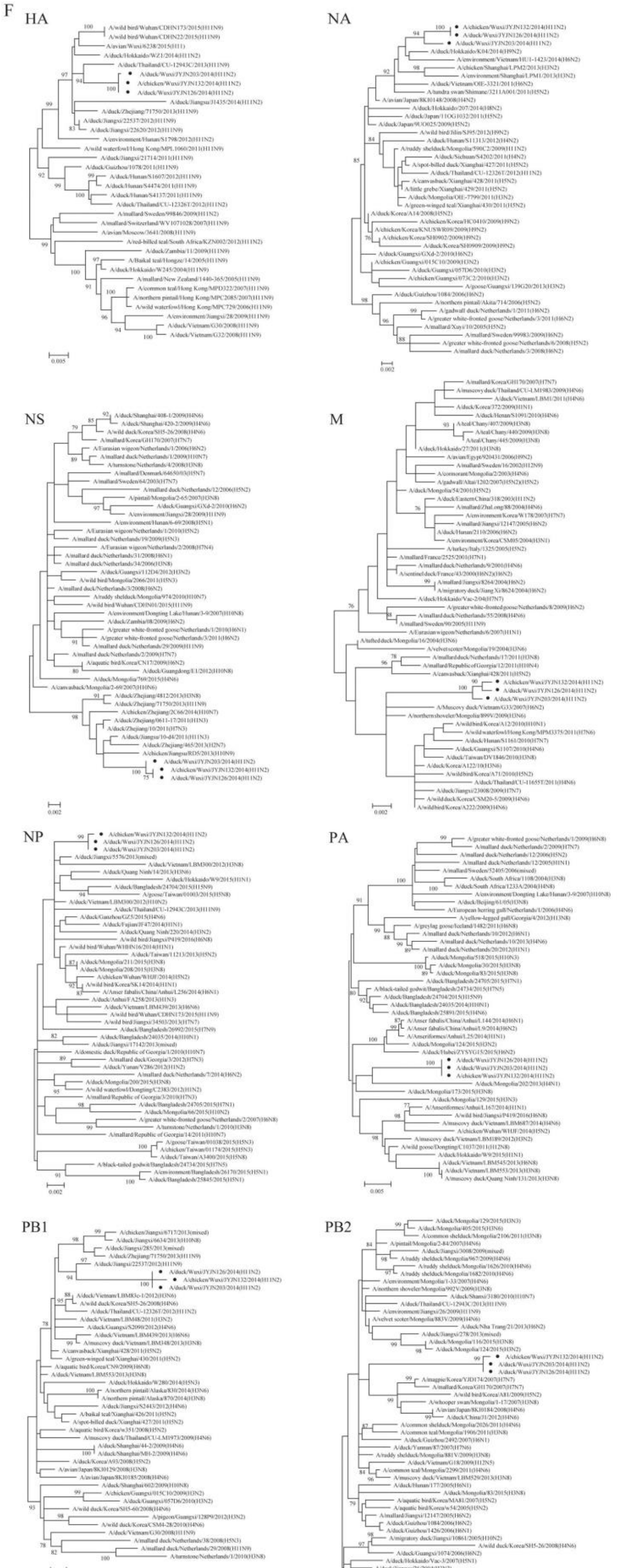
F
HA
NA
NS
M
NP
PA
PB1
PB2

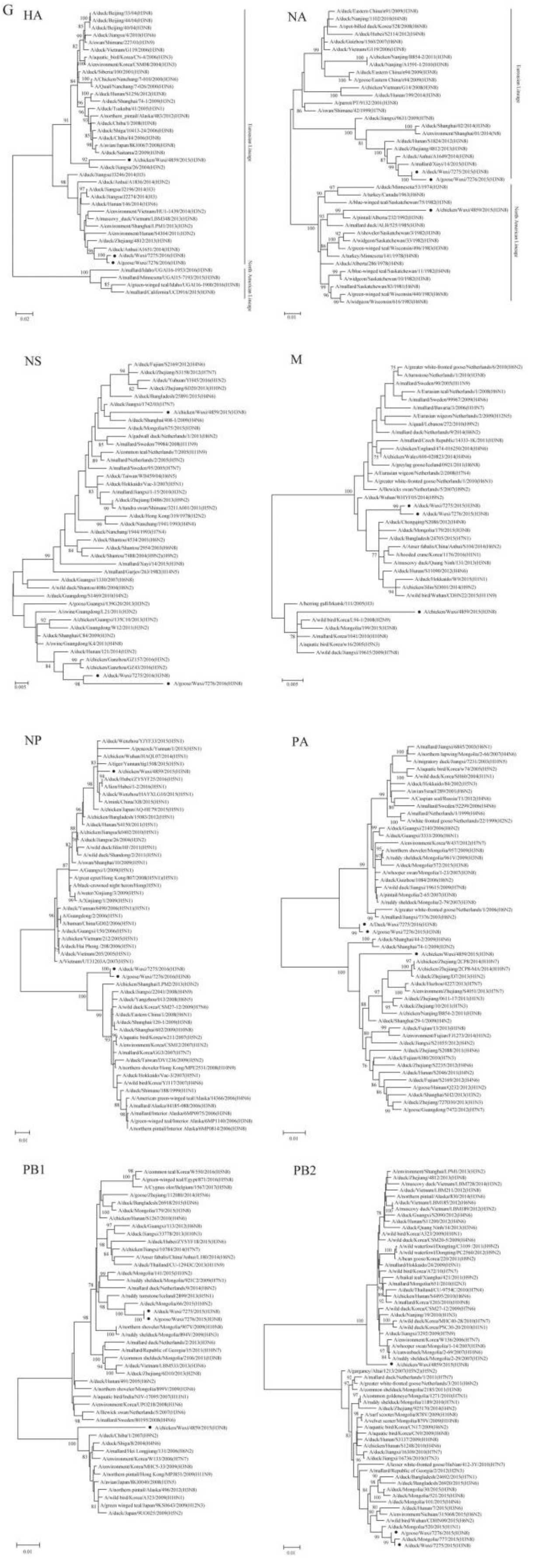
G
HA
NA
NS
M
NP
PA
PB1
PB2

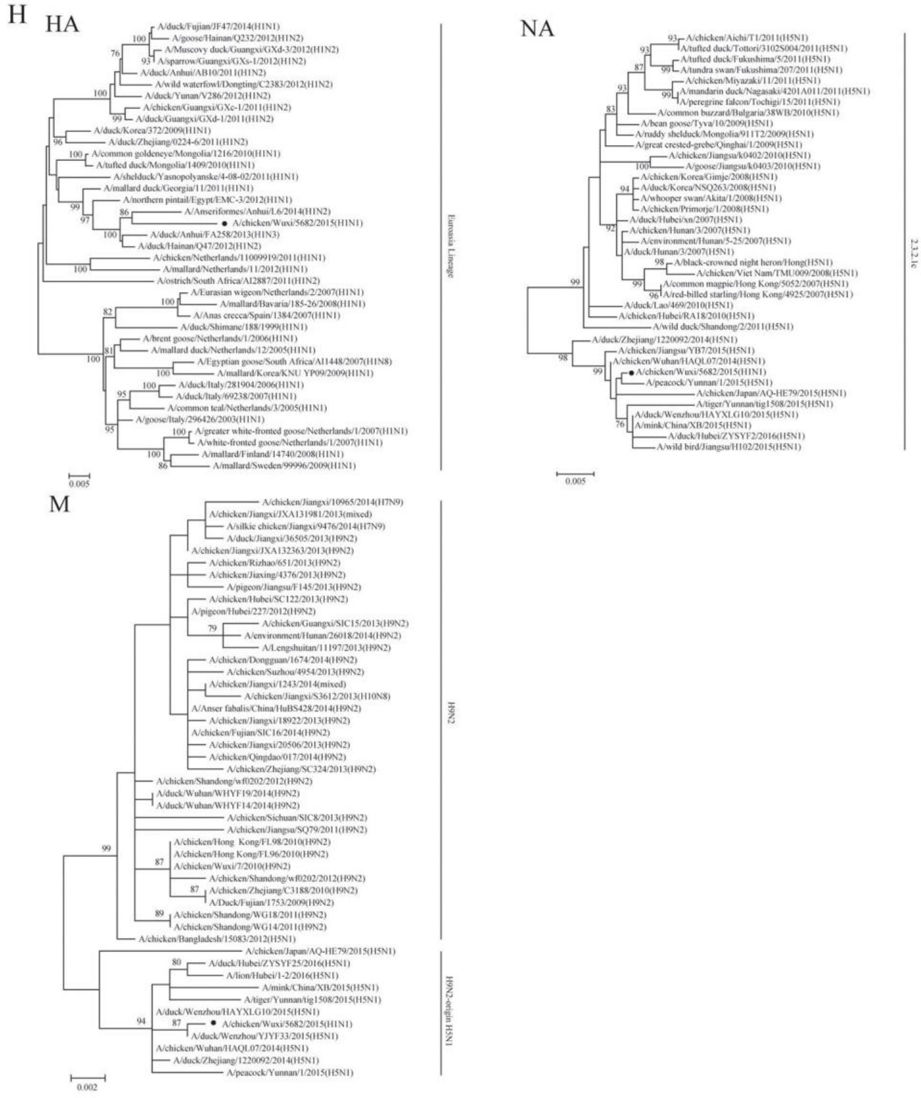

Technical Appendix Figure. Maximum-likelihood phylogenetic tree of influenza A viruses identified in the present study, Wuxi City, Jiangsu Province, eastern China, 2013–2016. The phylogenetic trees of the H7N9 (A), H9N2 (B), H5N6 (C), H5N1 (D), H5N2 (E), H11N2 (F), H3N8 (G), and H1N1 (H) subtypes are shown in panels A–H successively. Bootstrap values (n = 1,000) at key nodes are indicated. Values <75 were not shown. Scale bar indicates evolutionary distance (nucleotide substitutions per site). Black dots indicate the viruses reported in this study. Black triangles indicate the viruses had been previously reported. HA, hemagglutinin; NA, neuraminidase; NS, nonstructural; M, matrix; NP, nucleoprotein; PA, polymerase acidic; PB1, polymerase basic 1; PB2, polymerase basic 2.
